# Identifying T Cell Receptors from High-Throughput Sequencing: Dealing with Promiscuity in TCR*α* and TCR*β* Pairing

**DOI:** 10.1371/journal.pcbi.1005313

**Published:** 2017-01-19

**Authors:** Edward S. Lee, Paul G. Thomas, Jeff E. Mold, Andrew J. Yates

**Affiliations:** 1 Institute of Infection, Immunity & Inflammation, Glasgow Biomedical Research Centre, University of Glasgow, Glasgow, United Kingdom; 2 St. Jude Children’s Research Hospital, Memphis, Tennessee, United States of America; 3 Karolinska Institute, CMB, Stockholm, Sweden; La Jolla Institute for Allergy and Immunology, UNITED STATES

## Abstract

Characterisation of the T cell receptors (TCR) involved in immune responses is important for the design of vaccines and immunotherapies for cancer and autoimmune disease. The specificity of the interaction between the TCR heterodimer and its peptide-MHC ligand derives largely from the juxtaposed hypervariable CDR3 regions on the TCR*α* and TCR*β* chains, and obtaining the paired sequences of these regions is a standard for functionally defining the TCR. A brute force approach to identifying the TCRs in a population of T cells is to use high-throughput single-cell sequencing, but currently this process remains costly and risks missing small clones. Alternatively, CDR3*α* and CDR3*β* sequences can be associated using their frequency of co-occurrence in independent samples, but this approach can be confounded by the sharing of CDR3*α* and CDR3*β* across clones, commonly observed within epitope-specific T cell populations. The accurate, exhaustive, and economical recovery of TCR sequences from such populations therefore remains a challenging problem. Here we describe an algorithm for performing frequency-based pairing (alphabetr) that accommodates CDR3*α*- and CDR3*β*-sharing, cells expressing two TCR*α* chains, and multiple forms of sequencing error. The algorithm also yields accurate estimates of clonal frequencies.

## Author Summary

Our repertoires of T cell receptors (TCR) give our immune system the ability to recognise a huge diversity of foreign and self antigens, and identifying the TCRs involved in infectious disease, cancer, and autoimmune disease is important for designing vaccines and immunotherapies. The majority of T cells express a TCR made up of two chains, the TCR*α* and TCR*β*, and high-throughput sequencing of samples of T cells results in the loss of this pairing information. One can identify TCR*αβ* clones using single-cell sequencing, but this is costly and typically probes only part of the diversity of T cell populations. Statistical approaches are potentially more powerful by sequencing the TCR*α* and TCR*β* in multiple samples of T cells and pairing them using their frequency of co-occurrence. However, T cells involved in immune responses frequently share TCR*α* and TCR*β* chains with other responding cells. This promiscuity, combined with a high prevalence of T cells with two TCR*α* chains and sequencing errors, presents significant challenges to frequency-based pairing methods. Here we present a new algorithm that addresses these challenges and also provides accurate estimates of the abundances of T cell clonotypes, allowing us to build a more complete picture of T cell responses.

## Introduction

The ability of T cells to recognise antigens is conferred by a process of gene rearrangement that generates a diverse repertoire of T cell receptors (TCR), or clonotypes. Identifying the clonotypes involved in responses against pathogens and tumours or those involved in autoimmune disease can guide the design of vaccines and immunotherapies. In addition, the breadth of a T cell response correlates positively with the efficiency of control in many viral infections [1–3]. Thus, a method to characterise the diversity of antigen-specific responses—that is, the participating TCRs and their relative abundances—may yield potential correlates of protection.

The *αβ* TCR is a heterodimer, generated by a combination of ordered recombination of V, D, and J gene segments for the *β* chain and V and J gene segments for the *α* chain, together with random nucleotide insertions and deletions between the gene segments. The hypervariable CDR3*α* and CDR3*β* regions contact the peptide-loaded MHC (pMHC) most closely and so are considered the primary source of specificity in binding. From hereon we will use the term ‘chain’ interchangeably with the CDR3 region of the TCR*α* or TCR*β*. Historically, the CDR3*β* has been thought to contribute more to the interaction with pMHC due to its greater theoretical diversity. However, studies of crystal structures have demonstrated that CDR3*α* loops can have equal or greater contact with pMHC, as measured by buried surface area [4]. Epitope-specific immune responses also show biases for certain V and J segments in both *α* and *β* chains [5, 6], suggesting both chains contribute to the binding affinity. The *α* chain may even play a dominant role in the recognition of certain antigens [7]. Characterising the true extent of clonal diversity within T cell populations therefore requires resolving the paired CDR3*α* and CDR3*β* sequences within them.

Standard methods of multiplex PCR and high-throughput sequencing lose this pairing information and as a result are commonly used to analyze either the *α* or *β* chains alone [8–11]. More recent studies have used single-cell sequencing approaches to identify TCR*αβ* pairs, and, analogously, the paired CDR3 sequences from the heavy and light chains of the B cell receptor. These approaches include using single-cell sorting and RT-PCR [12–14], also with barcoding [15–18]; and variations of emulsion techniques to isolate single cells and amplify with PCR [18–20]. Drawbacks of these techniques include limited scalability, the risk of undersampling rare clones and so underestimating diversity, imprecise information regarding clonal abundances, and the need to use customised equipment [18, 21].

An alternative strategy is to use statistical methods to associate the CDR3*α* and CDR3*β* sequences obtained from bulk sequencing of multiple subsamples of T cells taken from the parent population of interest [22]. This approach exploits the fact that paired chains will tend to appear together in samples and uses the frequencies of these co-occurrences to associate them. A similar approach has been used to pair the heavy and light chains of B cells [23]. Because frequency-based pairing can be applied to large samples of cells, it has the potential to recover antigen receptors in greater depth and more economically than single-cell approaches, as well as providing more precise estimates of clonal frequencies. However, several properties of antigen-specific T cell populations present difficult challenges to this method. First, there is accumulating evidence from single-cell sequencing studies that, within an individual, T cell clonotypes specific for a given pMHC can exhibit sharing of both *α* and *β* chains [13, 14, 17, 19]. Second, between 10–30% of T cells possess two productive *α* chains [13, 24, 25] and 6–7% of T cells possess two productive *β* chains [25, 26]. The combination of sharing of *α* or *β* chains, dual TCRs, and sequencing errors can confound frequency-based methods that assume unique pairings. To illustrate, frequent co-occurrences of the three chains *α*_1_*α*_2_*β* in samples may derive from a single clone possessing two *α* chains or two clones *α*_1_*β* and *α*_2_*β* present at similar abundances, and the two possibilities are difficult to distinguish.

Here we describe a novel approach to frequency-based pairing that addresses these issues and identifies TCR*αβ* clones and their relative abundances using high-throughput sequencing of CDR3*α* and CDR3*β* regions. Our approach is optimised for antigen-specific populations and designed for use with cells recovered from typically-sized human blood samples. It is specifically designed to deal with promiscuity in *αβ* pairing, dual TCR*α* clones, and high rates of sequencing errors. By drawing on bulk sequencing data, we increase the efficiency of detection of rare responding clones and reduce the costs associated with single-cell high-throughput sequencing methods. The method also goes beyond other currently available approaches, yielding estimates of the frequencies of clones within their parent populations.

## Results

### Sharing of TCR*α* and TCR*β* chains across epitope-specific clones within an individual is common

Performing frequency-based pairing is in principle relatively straightforward if each clone is identified by two unique TCR*α* and TCR*β* chains. However, single-cell analyses of epitope-specific T cell populations in mice and humans have revealed significant levels of sharing of both CDR3*α* and CDR3*β* sequences at the amino acid level across clones within individuals (Table 1).

**Table 1:** A summary of the degrees of sharing of CDR3*α* and CDR3*β* at the amino acid level across clones within epitope-specific T cell populations, found in published single-cell TCR sequencing data and our own. Unless indicated otherwise, the samples were obtained from influenza-infected mice. The data clearly demonstrate that sharing of both *α* and *β* chains within an individual occurs in different infection/inoculation settings.

Citation	Peptide/System	Status	Number of clones	Number of distinct *α* chains	Number of distinct *β* chains	Number of shared *α* chains	Number of shared *β* chains	% of *α* chains that are shared	% of *β* chains that are shared
[13]	K^b^PB1_703_	Immune	35	16	24	3	2	18.8	8.3
[17]	Human CD4^+^ TILs	Colon cancer	216	226	216	7	0	3.1	0.0
	CD4^+^ T cells from adjacent colon		305	239	237	15	0	6.3	0.0
[14]	D^b^NP_366_	Naive 1	17	17	15	0	2	0.0	13.3
		Naive 2	11	11	11	0	0	0.0	0.0
		Naive 3	7	7	7	0	0	0.0	0.0
		Naive 4	10	7	9	3	1	42.9	11.1
		Naive 5	13	13	12	0	1	0.0	8.3
		Naive 6	9	9	9	0	0	0.0	0.0
		Immune 1	12	10	8	2	3	20.0	37.5
		Immune 2	15	9	8	4	3	44.4	37.5
		Immune 3	12	11	8	1	1	9.1	12.5
		Immune 4	10	10	8	0	1	0.0	12.5
	D^b^PA_244_	Naive 1	11	11	11	0	0	0.0	0.0
		Naive 2	10	10	10	0	0	0.0	0.0
		Naive 3	8	8	8	0	0	0.0	0.0
		Naive 4	25	25	25	0	0	0.0	0.0
		Naive 5	43	40	43	2	0	5.0	0.0
		Immune 1	17	15	15	2	1	13.3	6.7
		Immune 2	27	21	20	5	6	23.8	30.0
		Immune 3	14	14	12	0	2	0.0	16.7
		Immune 4	20	14	20	3	0	21.4	0.0
	D^b^PB1-F2_62_	Naive 1	13	13	13	0	0	0.0	0.0
		Naive 2	13	12	13	1	0	8.3	0.0
		Naive 3	9	9	9	0	0	0.0	0.0
		Naive 4	41	41	41	0	0	0.0	0.0
		Naive 5	21	21	21	0	0	0.0	0.0
		Naive 6	24	22	23	2	1	9.1	4.4
		Naive 7	16	16	16	0	0	0.0	0.0
		Immune 1	9	9	8	0	1	0.0	12.5
		Immune 3	11	11	11	0	0	0.0	0.0
		Immune 4	20	15	17	1	2	6.7	11.8
		Immune 5	16	15	16	1	0	6.7	0.0
This study	Human CD8^+^ YFV	Immune	184	169	179	15	3	8.9	1.7

The current upper limits on estimates of the number of unique TCR*β* chains in the naive CD4 or CD8 pools are 10^6^ in mice [27] and 10^8^ in humans [28]. As a consequence, sequencing of samples of naive T cells typically results in nearly every cell possessing a unique TCR*β* (see S1 Text, Section 1). Nevertheless, the true diversity of the naive repertoire may be even greater; due to the sequence of events involved in the generation of the TCR in the thymus, we expect each TCR*β* to be shared with many TCR*α* within the naive CD4 and CD8 T cell pools. In mice, thymocytes undergo 6–9 divisions following TCR*β* rearrangement at the DN3 stage [29–32], generating 64–512 cells which then undergo independent TCR*α* rearrangements. Assuming 5% of these TCR*αβ* precursors survive selection [33–36] leaves TCR*β* clone sizes of 3–25 cells post-selection [27]. Thymocytes may undergo 1 or 2 divisions at the single-positive CD4 or CD8 stage before leaving the thymus [36]; if we assume a 2-fold expansion here on average, each *αβ* T cell precursor at DN3 generates 6–50 new naive cells with identical TCR*β* chains, comprising 3–25 unique TCR*αβ* clones of typically 2 cells. Comparable estimates of TCR*β* clone sizes have been obtained elsewhere [27, 32]. There is also evidence that TCR*β*-clone sizes can be augmented by convergent recombination of the TCR*β* chain [8, 37]. If a particular CDR3*β* contributes strongly to the affinity of binding to a given peptide-MHC, then because the recruitment of naive antigen-specific T cells appears to be highly efficient [38], our rough quantification of TCR*αβ* clonality in thymopoesis is consistent with the observation that TCR*β*-sharing is commonly found within epitope-specific populations (Table 1).

**Fig 1:**
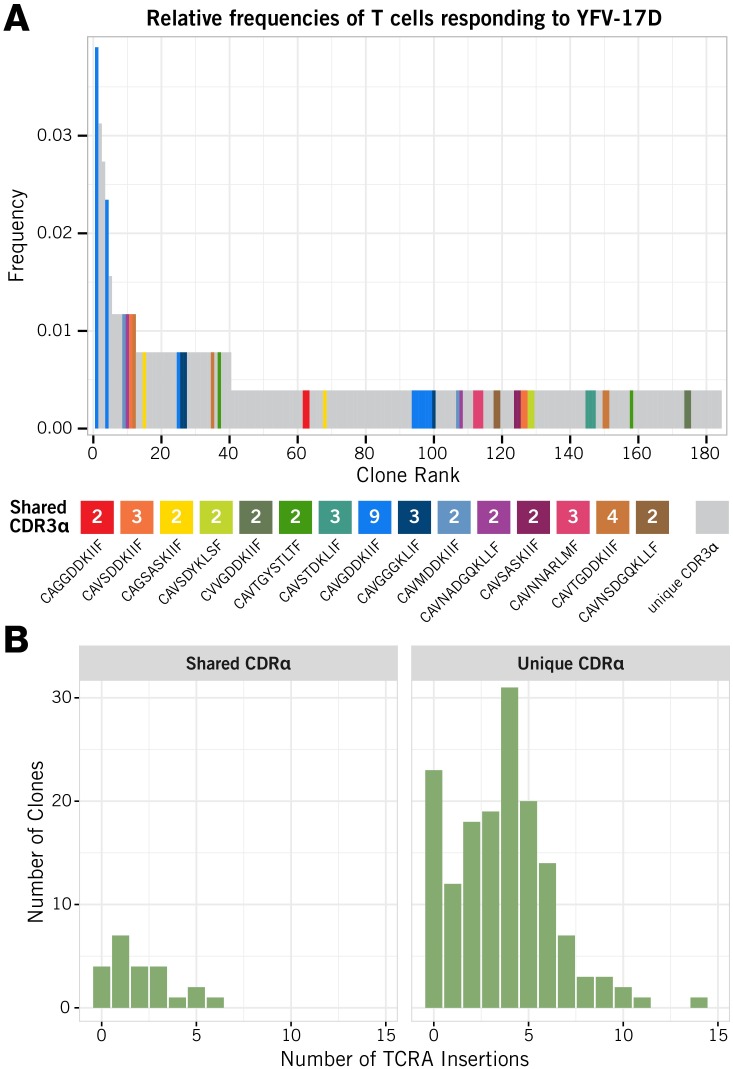
Analysis of TCR*α* usage in human, YFV-specific peripheral-blood CD8^+^ T cells. **(A)** Observed distribution of relative clone sizes within the population specific for the HLA-A02:01/LLWNGPMAV epitope. Clones expressing a unique CDR3*α* are shown in grey; clones that share a CDR3*α* are coloured, and the numbers in the coloured boxes represent the number of clones sharing each CDR3*α*. **(B)** The distributions of CDR3*α* nucleotide insertion lengths in clones with shared CDR3*α* (left hand panel) and unique CDR3*α* (right hand panel).

Because the rearrangement of the TCR*α* follows that of the TCR*β*, any sharing of CDR3*α* sequences across clones presumably arises from convergent recombination. Sharing then would be expected to arise most frequently for sequences that are close to germline, containing relatively few random N-nucleotide insertions. To examine this possibility, we immunised an HLA-A2 human volunteer with the live attenuated yellow fever vaccine YFV-17D, took a peripheral blood sample 15 days post-vaccination, and used dextramer staining and single-cell RNAseq to recover paired TCR*αβ* sequences from CD8^+^ T cells specific for the immunodominant epitope HLA-A02:01/LLWNGPMAV (see Methods; data provided in S1 Dataset). Out of 256 cells, we observed 169 unique CDR3*α*, with 15 (8.9%) of them shared between two or more clones (Fig 1A). We examined the numbers of nucleotide insertions at the V-J junction of the CDR3*α* and indeed saw significantly fewer in CDR3*α* sequences that were shared between two or more clones (mean 2.04 insertions, *n* = 23) than in sequences that were unique to a single clone (mean 3.62 insertions, *n* = 154; *p* < 0.005, Wilcoxon rank sum test; Fig 1B). In summary, it appears that convergent TCR*α* recombination may derive at least in part from the reduced junctional diversity of clones possessing CDR3 regions that are closer to germline.

### Experimental overview and computational approach

**Fig 2:**
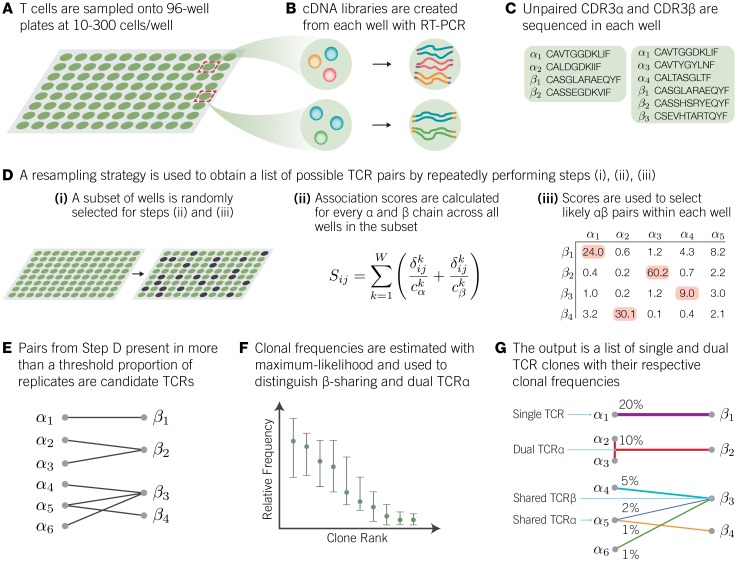
An overview of the implementation of alphabetr. **(A)** From the population of interest, multiple samples of 10–300 T cells are sorted into 96-well plates. This design allows for a given clone to be sampled in multiple wells. **(B)** Multiplex RT-PCR is used to create cDNA libraries of CDR3*α* and CDR3*β* from each well, and **(C)** high-throughput sequencing is used to recover the unpaired CDR3*α* and CDR3*β* sequences of the clones sampled in each well. **(D)**(i) A random subset of the wells is chosen, (ii) association scores between every unique *α* and *β* found across the wells within this sample are calculated, and (iii) the set of unique *αβ* pairs that maximises the sum of association scores is identified using the Hungarian algorithm [39]. Step (iii) is illustrated for a particular set of CDR3*α* and CDR3*β* recovered from one well, as a matrix of association scores calculated across all wells in the subsample. **(E)** Steps D(i)-(iii) are repeated to generate a consensus list of pairs, filtering out candidates that appear rarely across replicates. **(F)** The frequencies of each remaining candidate *αβ* pair within the parent population are estimated using a maximum-likelihood approach, assuming only sharing (no dual TCR). Dual TCR*α* clones *α*_1_ *α*_2_ *β*_1_ are then distinguished from clones apparently sharing a TCR*β* chain (*α*_1_ *β*_1_ and *α*_2_ *β*_1_), by examining the patterns of co-occurrences of the three chains, and the frequencies of these clones are re-calculated. **(G)** The output of the algorithm is a list of single and dual TCR*α* clones, each with their estimated frequency within the parent population. See text and Methods for more details.

Motivated by this promiscuity of TCR*α* and TCR*β* pairings, we developed a semi-heuristic procedure alphabetr (**AL**gorithm for **P**airing alp**HA** and **BE**ta **T** cell **R**eceptors) that recovers TCR*αβ* pairs from high-throughput sequencing data. Fig 2 shows the algorithm schematically. The experimental procedure is to sequence the CDR3*α* and CDR3*β* regions from multiple samples of T cells from the same parent population (Fig 2A–2C). The input to the algorithm is a list of these unpaired sequences (Fig 2C), each associated with the sample it belonged to (e.g. a given well in one or more 96-well plates). Fig 2C illustrates amino acid sequences as inputs, but the algorithm can be applied equally well to data comprising nucleotide sequences and/or the addition of V(D)J segment information. The number of cells in each well can be freely varied, and indeed as we describe below, varying the sample size across the plate(s) helps to increase both the number and accuracy of pairings. Given this information, alphabetr then calculates association scores between every *α* and every *β* chain found in a randomly chosen subsample of wells. This score is the sum of the number of co-occurrences of chains in each well, each weighted inversely by the total number of chains recovered from that well (Fig 2D(ii)). The weighting factor reflects the intuitive idea that our confidence that a co-occurring *α* and *β* pair derive from the same clone decreases as the number of unique chains recovered from that well increases. The algorithm then solves a linear sum assignment problem within each well based on these plate-wide association scores to generate a list of candidate pairs of *α* and *β* sequences within each well (Fig 2D(iii)). This is a list of *αβ* pairs in which each *α* is paired with only one *β*, and vice versa, such that the sum of the association scores is maximised. After repeating this assignment for every well in the subset, we generate a matrix of dimensions *n* × *m* where *n* and *m* are the total numbers of unique *α* and *β* chains recovered across the plate(s), respectively, and whose entries are the number of times that each candidate pair *α*_*i*_ *β*_*j*_ (*i* ∈ {1…*n*}, *j* ∈ {1…*m*}) have been associated. Sharing of chains across clones is now possible in this list. Those *αβ* pairs that appear in a number of wells greater than the mean of the non-zero elements of this matrix are retained as a refined list of candidate pairs. The pairing and filtering process is repeated on subsets of the data (Fig 2D), and a consensus list of putative paired CDR3 sequences comprises those appearing in more than a threshold proportion of these lists (Fig 2E). This pseudo-jacknife procedure acts to reduce the effect of very common clones pushing up the threshold for inclusion in the filtered list and increases the efficiency of pairing of rarer clones, while minimising the inclusion of incorrect *αβ* pairs. Steps A-D are described in more detail in Methods.

The algorithm then uses a maximum likelihood approach to estimate the relative frequencies of the clones associated with each candidate *αβ* pair (Fig 2F; Methods). These estimated frequencies are then used with the patterns of co-occurrences of chains to distinguish between *β*-sharing and dual TCR*α* clones (see Methods). This step also yields refined estimates of the frequencies of dual TCR*α* clones. The output of the algorithm is a list of single or dual TCR*α* clones together with estimates of their abundances within the parent population (Fig 2G).

### Testing on synthetic datasets

To test the performance of alphabetr, we first used artificially generated datasets mimicking the bulk sequencing of CDR3*α* and CDR3*β* regions from polyclonal T cell populations. We assumed skewed distributions of clone sizes, with between 5 and 50 clones comprising the most abundant 50% of the population and the remainder, approximately 2000 clones, forming a flat tail at low frequency (see Methods). These distributions were chosen to reflect plausible immunodominance hierarchies within T cell responses, motivated by analysis of epitope-specific cells recovered from human subjects immunised with live attenuated yellow fever virus vaccine (our analysis and ref. [11]). We also analysed different sizes of parent populations (see S1 Text, Section 2). Within these hierarchies we allowed the virtual clones to exhibit sharing of CDR3*α* and CDR3*β* at ranges of frequencies consistent with published single-cell TCR sequencing studies (Table 1) and our own data (Fig 1A). We also allowed between 10% and 30% of clones to express two productive TCR*α* chains and 6% of clones to express two productive TCR*β* chains. The sequences in each ‘well’ were then generated by sampling between 10 and 300 T cells from the parent population with replacement. Selecting an optimal pattern of sampling is an issue we return to below.

To assess the robustness of alphabetr, we simulated the properties of two forms of sequencing error: dropping of chains and productive in-frame sequencing errors. Dropping of chains represents the failure of CDR3*α* and/or CDR3*β* regions to amplify or be detected, a process which likely has both purely random and clone-specific elements [22]. To model this, each clone was assigned a drop rate at random from a lognormal distribution with mean 0.15 and standard deviation of 0.01, with the rate capped at 0.9. Each instance of a CDR3*α* and CDR3*β* from that clone was then removed from the well with probability equal to the drop rate. To model productive in-frame sequencing errors, every unique CDR3*α* and CDR3*β* was assigned an error rate randomly drawn from a lognormal distribution with mean 0.02 and standard deviation 0.005. Each instance of a sequence at the per-cell level was replaced at random by one of three erroneous ‘daughter’ sequences, unique and specific to the parent sequence, with probability equal to the sequence-specific in-frame error rate. Thus on average each CDR3*α* and CDR3*β* generated mutant offspring sequences at the rate of 2% per instance in each cell in the plate(s).

We then assigned identifiers to the remaining CDR3*α* and CDR3*β* sequences, associating them with the sample’s location in a virtual 96-well plate. The input to the algorithm is the list of these unpaired CDR3*α* and CDR3*β* sequences together with their well-identifiers. This process was repeated for different sampling strategies (varying the sample sizes within each well, and using one or five 96-well plates); different clonal size distributions; and different degrees of CDR3*α* and CDR3*β* sharing. Under these ranges of conditions, the algorithm was tested for the following:

1. *Overall depth*, the number of *αβ* pairs that were correctly identified, as a proportion of the total number in the parent population (here a dual TCR*α* clone *α*_*j*_ *α*_*k*_ *β* is treated as two clones *α*_*j*_ *β* and *α*_*k*_ *β*—see points 4 and 5)
2. *Depth of top clones*, the proportion of those clones that comprise 50% of the population after ranking by abundance that were correctly identified
3. *False pairing rate*, the proportion of identified *αβ* pairs that were incorrect
4. *Adjusted dual depth*, a measure of how well dual TCR*α* clones can be identified from candidate pairs:
  $$=\frac{\#\text{ correctly identified dual TCR}\alpha \text{ clones}}{\#\text{ true dual TCR}\alpha \text{ clones whose two }\alpha \text{ chains are in the list of candidate }\alpha \beta \text{ pairs}}$$
5. *False dual rate*, the proportion of candidate dual TCR*α* clones that were incorrectly identified.

alphabetr does not attempt to identify dual TCR*β* expressing cells because dealing with this relatively infrequent phenomenon together with dual TCR*α* chains and sharing of both TCR*α* and TCR*β* chains across clones is extremely challenging algorithmically. However, we include dual TCR*β* cells in our simulated data at the level of 6% to establish their impact on the algorithm’s performance.

#### A mixed sampling strategy with stringent co-incidence criteria strikes a balance of depth and accuracy of pairing

**Fig 3:**
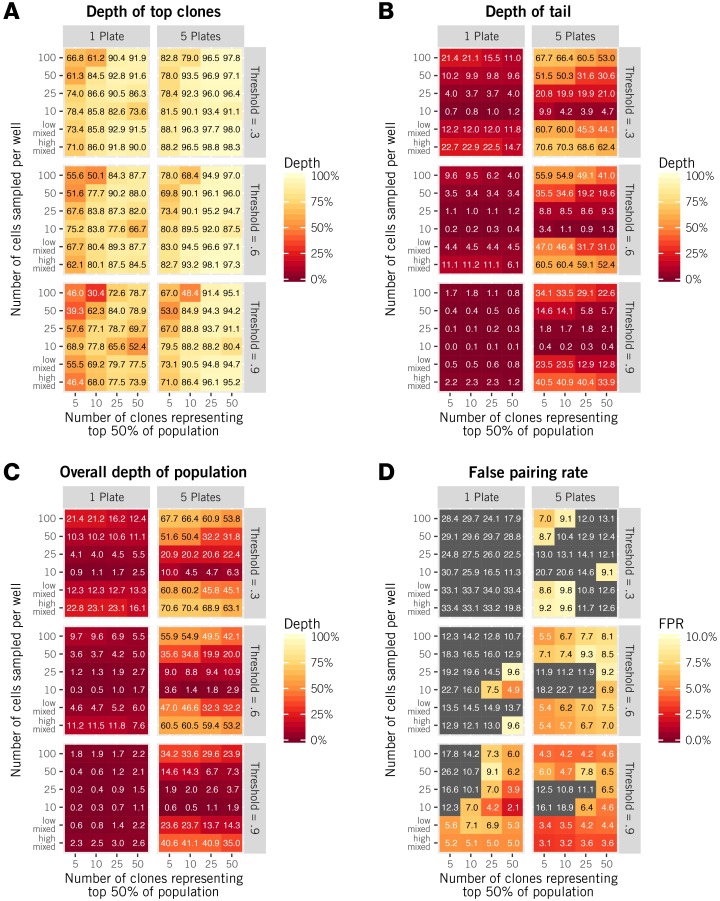
Depth and accuracy of *αβ* pairings generated by alphabetr, for a range of overall sample sizes, sampling strategies and underlying distributions of clone sizes. Simulations were performed using *in silico* data sets of one or five plates using six different sampling strategies (see text) and different degrees of skewness in clonal frequencies, as indicated by the number of clones comprising 50% of the population when ranked by frequency. ‘Threshold’ refers to the stringency of pair association, *T* (see Methods). **(A)** The proportion of the most abundant 50% of clones that were identified. **(B)** The proportion of the least abundant 50% of clones that were identified. **(C)** The overall depth was influenced strongly by the tail depth, indicating that data from one plate may be sufficient for recovering the most common clones. **(D)** The rate at which CDR3*α* and CDR3*β* sequences were incorrectly paired (false positive rate, FPR).

Fig 3 shows the depth and accuracy of pairing using simulated data. These were generated by sampling from parent populations of 2100 clonotypes exhibiting sharing of both TCR*α* and TCR*β* chains, with drop rates and in-frame error rates of the CDR3*α* and CDR3*β* sequences drawn from lognormal distributions as described above. To test the algorithm robustly, we assumed 30% of clones expressed two TCR*α*, a prevalence at the upper limit of estimates from the literature [24]. We tested the ability of the algorithm to associate CDR3*α* and CDR3*β* sequences for different distributions of clonal frequencies and for different sampling strategies, using fixed numbers of cells per well (10, 25, 50, 100) or two mixed strategies (Table 2). We also show results for different degrees of consensus required for pair selection. For each set of conditions, performance metrics were computed by averaging the results of 100 simulated experiments.

**Table 2:** The mixed sampling strategies used in the simulations.

Sampling Strategy	Number of plates	Number of wells × number of cells per well					
High-Mixed	1	26 × **20**	13 × **50**	19 × **100**	19 × **200**	19 × **300**	
	5	128 × **20**	64 × **50**	96 × **100**	96 × **200**	96 × **300**	
Low-Mixed	1	26 × **15**	6 × **20**	13 × **30**	19 × **50**	19 × **100**	19 × **150**
	5	96 × **15**	32 × **20**	64 × **30**	96 × **50**	96 × **100**	96 × **150**

With only a single plate, the most abundant 50% of clones can be recovered with depths between 62% and 89% with a moderate threshold of 0.6 and the mixed sampling strategies, improving with less skewed distributions (Fig 3A). Coverage of rare clones (Fig 3B, left panels) is much more limited, particularly—and unsurprisingly—for sparse sampling strategies, but improves with a more lenient consensus threshold of 0.3. Using five plates boosts the recovery of rare clones considerably (Fig 3B, right panels), providing up to 61% depth with a threshold of 0.6 and 70% with a threshold of 0.3. As a result, for all sampling strategies, the effect of increasing the number of plates—and hence total sample size—is to increase overall depth (Fig 3C), almost entirely through greater recovery of rarer clones.

Increasing the number of plates also significantly reduces the false pairing rate (Fig 3D), which can be as low as 3.1% for 5 plates and a stringent threshold of 0.9 (Fig 3D, lower right panel). In general, and as expected, increasing the stringency threshold reduces false pairing rates. However, the stringency of the threshold can be relaxed if there is no significant presence of dual TCR*β* clones in the T cell population of interest (S1 Text, Section 2 and Fig F).

Increasing the stringency (threshold) of the pseudo-jacknife procedure—that is, requiring a high frequency of occurrence of candidate pairs across subsets of the data—results in a lower false pairing rate at the cost of lower depth, largely for rarer clones (Fig 3C and 3D). This is because rarer clones will be excluded from the jacknife replicates more often than common ones; as the stringency of pair selection is increased, rare clones will therefore tend to be filtered out.

In summary, mixed sampling strategies with moderate to high acceptance thresholds yield the lowest false pairing rates (Fig 3D) while maintaining good depth of recovery of rare clones (Fig 3B). The high-mixed strategy requires a larger overall sample size and thus achieves greater depths, particularly of rare clones.

#### Sampling strategies for epitope-specific T cells may be constrained by the ability to recover sufficient cells

In practice, the availability of cells may place constraints on the sampling strategy. For example, with five plates the high- and low-mixed strategies require a total of 64,000 and 33,000 cells respectively. A typical sample of four tubes (approximately 30ml) of human blood yields roughly 3 × 10^7^ PBMCs, of which roughly half are *αβ* T cells. With such a sample, numbers of T cells specific for immunodominant epitopes of highly immunogenic infections such as Epstein-Barr virus and cytomegalovirus [40–43], numbers are unlikely to be limiting. A conservative estimate is that to acquire 100,000 cells with which to implement the high-mixed sampling strategy on five 96-well plates requires epitope-specific frequencies in excess of 1% of *αβ* T cells, or 0.5% of PBMC. Frequencies below this may dictate fewer plates and/or a sparser sampling strategy (S1 Text, Section 2).

#### Exploring different degrees of TCR*α*- and TCR*β*-sharing, richness in clonal structure, and prevalence of dual TCR*β*

Our simulation approach allowed us to explore other plausible datasets. We simulated populations exhibiting sharing at the high and low ends of the levels quoted in the literature, as well as different levels of clonal diversity (S1 Text, Section 2). For mixed sampling strategies with five plates, higher sharing levels increased the false pairing rate by at most 4% in absolute terms, although the magnitude of this effect decreased as the stringency of pair selection was increased (S1 Text, Fig B). Lower levels of sharing decreased the false pairing rate by approximately 1% in absolute terms (S1 Text, Fig C). In both cases, the depths of recovery were very similar to those presented in Fig 3.

Simulations of populations with higher diversity (3000 clones) show similar false pairing rates, similar top depths, and slightly lower tail depths to those for 2000 clones, while simulations of populations with 500 clones show slightly lower top depths, higher tail depths, and higher false pairing rates (S1 Text, Fig D and E). Populations comprising fewer clones overall will by definition display higher relative abundances, and as we discuss below, in such situations frequency-based pairing approaches will benefit from sparser sampling strategies.

Although alphabetr does not identify dual TCR*β* clones, we performed simulations to compare how the presence of such clones in the parent population affects the ability of alphabetr to associate TCR*α* and TCR*β* correctly (S1 Text, Fig F). The presence of dual TCR*β* clones at a frequency of 6% increases the false pairing rate by approximately 3% in absolute terms, while not affecting the top and tail depths. Since significant levels of dual TCR*β* clones have been shown in only a small number of studies sequencing antigen-specific T cell populations [25, 26], we believe this represents an upper bound on the effect of dual TCR*β* clones on the performance of alphabetr.

#### Precise estimation of frequencies of common clones benefits from sparse or mixed sampling strategies

The probability that all chains associated with a clone co-appear in a given number of wells can be calculated straightforwardly from the binomial distribution. We can then use maximum likelihood to estimate this clone’s abundance within the parent population (see Methods).

**Fig 4:**
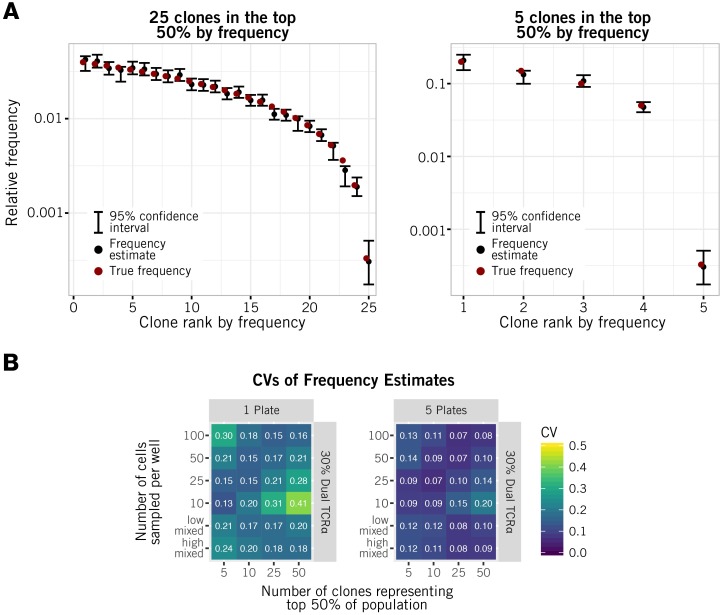
Assessment of the precision of clonal frequency estimation. **(A)** Point estimates of clonal abundances generated by alphabetr, derived from representative simulations using five plates and distributions with 25 and 5 clones in the top 50% (left and right panels respectively). (B) The coefficient of variation (precision) of abundance estimates for a range of skewnesses of clone sizes and sampling strategies. Values quoted are averages over 100 simulations.

We used this procedure to assess the ability of alphabetr to estimate clonal abundances over a range of clonal size distributions and sampling strategies (Fig 4). We show results only for the most abundant clones making up 50% of the population. The left and right panels of Fig 4A show typical sets of abundance estimates for populations with moderately and highly skewed clonal distributions, with 25 and 5 clones respectively making up the top 50% of clones by size. We tested the method of construction of point estimates and confidence intervals using simulated data and confirmed that close to 95% of such intervals contained the true frequency (results not shown).

Fig 4B summarises the precision of the abundance estimation for a variety of sampling strategies and skewnesses. We quote an approximate coefficient of variation (CV) $\hat{\sigma }/\hat{f}$, where $\hat{\sigma }$ is estimated using a quadratic approximation to the 95% confidence interval, 3.92$\hat{\sigma }$, and $\hat{f}$ is the estimated abundance. The procedure yielded CVs in the range 0.13–0.41 for one plate and 0.07–0.20 for five plates (Fig 4B).

Intuitively, the impact of skewness arises because we maximise the information regarding a given clone’s abundance when sample sizes are such that the clone appears in an intermediate proportion of wells. Sampling low numbers of cells is therefore optimal for determining the abundance of highly immunodominant clones, and larger numbers are optimal for determining the abundance of rare clones. For the clone distributions considered here, for common clones the sparsest sampling strategy (uniformly 10 cells/well) gives the greatest precision. In general, however a mixed sampling strategy strikes a balance between precision over a wide range of abundances (Fig 4B, bottom row in each panel), false pairing rates, and depth.

The clonal abundances shown in Fig 4A depend on prior knowledge or estimation of the mean drop rate, or the mean probability that any CDR3*α* or CDR3*β* of a clone will fail to be sequenced (see Methods). Neglecting this error rate yields lower bounds on clonal abundances (S1 Text, Section 3).

#### Efficient discrimination of dual TCR*α* and TCR*β*-sharing clones requires a mixed sampling strategy and distinct methods for common and rare clones

The final step in the algorithm is to decide whether each candidate pair of clones that share a *β* chain (e.g. *α*_1_*β* and *α*_2_*β*) are indeed two clones or derive from one clone with a dual TCR*α* (*α*_1_*α*_2_*β*). To do this, we exploit the fact that the patterns of co-occurences of all three chains will be different under the two hypotheses. Initially, we use the estimated frequencies of a putative *β*-sharing clone pair *α*_1_ *β* and *α*_2_ *β* to calculate the expected number of wells in which all three chains should co-occur. Essentially, the three chains will tend to co-occur more frequently if they derive from a dual TCR*α* clone than if they derive from two *β*-sharing clones. We construct the ratio of the expected to the observed numbers of three-way co-occurrences for each *β*-sharing pair and perform *k*-means clustering on these ratios. The cluster of higher values forms the first list of candidate dual TCR*α* clones. See Methods for details and S1 Text, Section 5 for a visual example of the clustering of clones into two groups.

However, performing *k*-means clustering on only the numbers of three-way occurrences is inefficient at discriminating *β*-sharing and dual TCR*α* clones that are relatively abundant because the expected frequencies of co-occurrences become indistinguishable, particularly for rich sampling strategies in which the three chains co-occur in many wells. We therefore added a second step which utilises more information from the plates, calculating the likelihoods of all three- and two-way concurrences of *α*_1_, *α*_2_ and *β* under both hypotheses. Exact computation of these likelihoods is only practical for the low-occupancy wells (less than 50 cells/well), which conveniently are also the wells that contain maximal information regarding common clones. As a result, this second approach can be performed only when using sparse sampling strategies or the low-occupancy wells used in the mixed sampling strategies. We determined empirically that differences in the log-likelihoods of more than 10 distinguish the *β*-sharing and dual TCR*α* hypotheses.

The ability of these procedures to identify dual TCR*α* clones depends on alphabetr associating both TCR*α* chains with the TCR*β* chains of these clones (e.g. associating *α*_1_ and *α*_2_ with *β* for a dual *α*_1_*α*_2_*β* clone). We therefore assess the efficiency of the discrimination using the ‘adjusted dual depth’—the number of correctly identified dual TCR*α* clones divided by the number of true dual TCR*α* clones whose constituent chains appeared in the candidate list of *αβ* pairs (that is, those dual TCR*α* clones *α*_1_*α*_2_*β* for which *α*_1_ and *α*_2_ were both paired with *β* in the first stage of the algorithm). We also calculate the false dual rate (FDR)—the proportion of the putative dual TCR*α* clones that were incorrectly identified.

**Fig 5:**
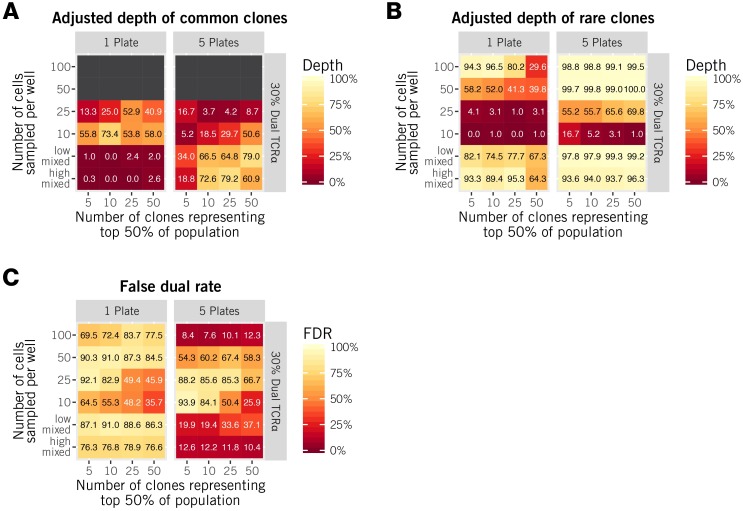
Discriminating between dual TCR*α* and *β*-sharing clones. We assess the degree of recovery of dual TCR*α* clones with the ‘adjusted depth,’ which is the proportion of dual TCR*α* clones correctly assigned out of the list of candidate dual TCR*α* and TCR*β*-sharing clones. Panel (A) shows the adjusted depth of common clones; panel (B), rare clones. For common clones, we used likelihood-based discrimination; for rare clones we used a clustering approach. Both procedures are detailed in Methods. Panel (C) shows the false dual rate averaged over all clones—the proportion of identified dual TCR*α* that are incorrect. All results are shown for a threshold of 0.3 with 30% prevalence of dual TCR*α* and are averages over 100 simulations.

Fig 5 summarises the ability of the algorithm to distinguish TCR*β*-sharing and dual TCR*α* clones. Common clones are identified through the three-way likelihood approach, and mixed sampling strategies give the best results in this case, with adjusted depths of up to 79% for less skewed distributions (Fig 5A). The likelihood approach still performs relatively poorly with very highly skewed populations, distinguishing dual TCR*α* from *β*-sharers correctly at most 34% of the time for population with 5 clones making up the top 50% of the population (Fig 5A). Under these circumstances, the statistics of co-incidence of the three chains are very similar under the two hypotheses of dual TCR*α* or TCR*β*-sharing clones. In contrast, the *k*-means procedure achieves adjusted depths of 93–99% for rare clones when using 5 plates and the high-mixed strategy (Fig 5B). Averaging over all clones, this strategy gives false dual rates of between 10–13% (Fig 5C).

#### Extensive single-cell sequencing is required to achieve equivalent overall depth to alphabetr

A key issue is whether implementing alphabetr improves upon single-cell approaches. One way to assess this would be to take a sample of antigen-specific cells, perform single-cell sequencing on a subset of these cells, and apply alphabetr to the remainder to compare their performance on the same set of parent clones. An alternative, which we perform here, is to simulate both scenarios. The advantages of the simulation approach are that it allows us to (i) triangulate both methods with the gold-standard of the true sequences, which are not known in practical settings due to dropping of chains and in-frame sequencing errors, and (ii) explore levels of single-cell sequencing that are currently prohibitively costly.

**Fig 6:**
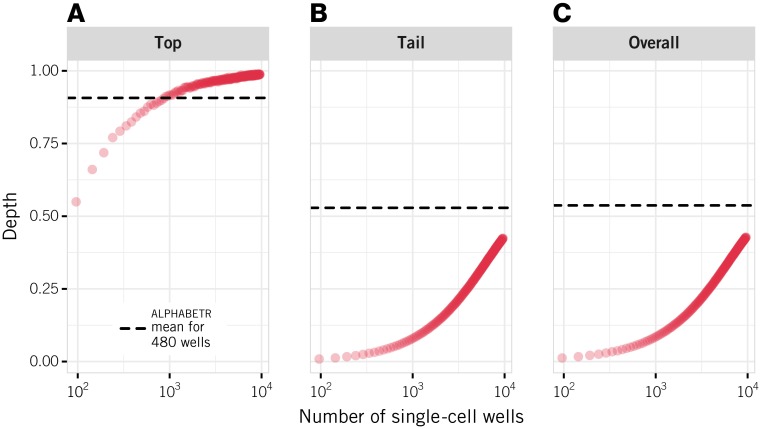
Comparison of single-cell approaches and alphabetr. Single-cell sequencing was simulated by sampling from the same populations used to evaluate alphabetr and including both the dropping of chains and in-frame sequencing errors. In these simulations, the parent population contains 2100 clones with 25 clones representing the top 50% of the clones ranked by abundance. The results were evaluated for (A) top depth, (B) tail depth, and (C) overall depth. The dashed lines show the mean performance of alphabetr applied to five plates using the high-mixed sampling strategy and a threshold of 0.6 (values taken from Fig 3). The single-cell sequencing results are averages of 200 simulations.

We simulated the sequencing of between 96 and 9600 single cells sampled from the same synthetic T cell populations used for evaluating alphabetr, and using the same model of sequencing errors. Fig 6 compares the performance of the two methods for a population of 2100 clones, with 25 clones making up the top 50% by abundance. alphabetr was implemented with the high-mixed sampling strategy of five plates and with a stringency threshold *T* = 0.6. We show performance comparisons using other distributions of clone sizes in S1 Text, Section 7. Under the conditions used for Fig 6, almost double the number of single-cell sequencing runs was required to achieve the same top depth yielded by alphabetr with five plates, and more than 100 plates of single cells are required to approach alphabetr’s level of recovery of rare clones. With the same clone size distribution, even a single plate analysed with alphabetr yields top depths from 78% to 92%, depending on the threshold parameter used (Fig 3A), whereas 96 single cells yield a top depth of 60% (Fig 6A). Single-cell sequencing will exhibit a false positive rate that is approximately twice the mean of the in-frame error rate, or 4% in our simulations, an accuracy that is comparable to that of alphabetr at its most stringent.

#### Applying alphabetr to real sequencing data

Using simulated data allowed us to assess the performance of alphabetr directly using the gold standard of known TCR*αβ* sequences and under a range of plausible experimental conditions. However, to illustrate a real-world application, we applied alphabetr to a published dataset derived from the TCRs of tumour-infiltrating lymphocytes (TILs) from human subjects [22]. The study also used a frequency-based method to pair the TCR*α* and TCR*β* obtained by sampling TILs from nine different tumours into the wells of one 96-well plate and sequencing the CDR3*α* and CDR3*β* chains found in each. One tumour (Breast 1) yielded only 7 pairs, and we excluded it from the analysis. We applied alphabetr to the chains from each of the remaining 8 tumours in turn. We then compared the pairs determined by alphabetr to those identified explicitly by ref. [22] (Table 3; see Section 8 of S1 Text for details). The true TCR clonotypes are unknown and so our aim was to measure degrees of concordance and conflict between the two methods. In 6 out of 8 tumours, alphabetr recovered fewer clones; however we found average concordance rates of 77%, defined as the proportion of the pairs identified by alphabetr that were also identified in ref. [22]. Perhaps more strikingly, we also found a very low incidence of conflicting pairs (mean 2% across tumours, as a proportion of all pairs identified by alphabetr). Conflicts were defined as those clones determined by the two methods that have only one chain in common.

**Table 3:** Recovery of tumour-infiltrating lymphocyte TCR pairs using alphabetr and data from ref. [22]. The data were processed by associating chains with their tumour sources through exact matching of the CDR3 nucleotide sequences from the mixed tumour samples to CDR3 libraries obtained from blood samples from each patient. The data were then simplified by selecting only those chains associated with one tumour. We then used alphabetr to identify TCR*αβ* pairs. The numbers of pairs unambiguously identified in ref. [22] were determined by directly matching nucleotide sequences to the CDR3 libraries, and only those pairs for which both chains could be directly associated with the corresponding tumour sample were included in the analysis.

Tumour Sample	Number of pairs identified by alphabetr	Number of pairs unambiguously identified by ref. [22]	Number of identical identified pairs	Percentage of alphabetr pairs agreeing with ref. [22]	Number of conflicting pairs	Number of novel pairs from alphabetr
Breast 2	98	85	74	75.5%	0	24
Breast 3	109	129	94	86.2%	1	14
Breast 4	50	85	26	52.0%	1	23
Kidney 1	74	112	58	78.4%	3	13
Kidney 2	145	286	126	86.9%	5	5
Kidney 3	213	282	166	77.9%	8	39
Kidney 4	157	176	131	83.4%	1	25
Lung 1	173	163	124	71.7%	1	48

**Fig 7:**
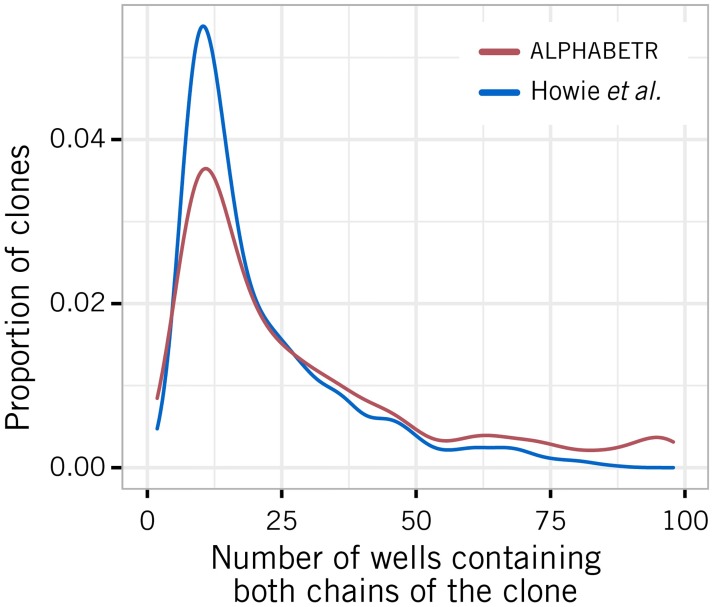
Comparison of well occupancy patterns of the clones identified by alphabetr and in ref. [22]. For each method, TCR*αβ* pairs identified for all tumour samples were combined to estimate the distribution of the number of wells in which the chains co-appeared. The differences between these distributions indicate the relative efficiency with which the two algorithms identify clones, as a function of their abundance.

To compare the abilities of the two algorithms to identify rare or common clones, we stratified the identified *αβ* chain pairs by the frequency with which they co-appeared in wells. With stringency thresholds greater than 0.7, we find that with a single 96-well plate and a sampling strategy optimised for use by the algorithm described in ref. [22], alphabetr is less efficient at identifying rare clones but identifies clones with moderate to high abundances—for which the TCR*α* and TCR*β* chains co-appear in more than a quarter of the wells—more efficiently (Fig 7; see Fig J in S1 Text for a breakdown by tumour). The clones identified by alphabetr alone exhibit moderate levels of sharing (TCR*α*-sharing, mean 16%, range 0–60%; TCR*β*-sharing, mean 13%, range 4–31%). Of the sharers, an average of 76% share a chain with a clone that was identified by both methods.

## Discussion

Applying high throughput single-cell sequencing technologies to very large numbers of T cells is becoming increasingly within reach, but smaller-scale solutions using frequency-based sampling potentially remain far more economical. While another implementation of this strategy exists [22], the promiscuous nature of TCR*α* and TCR*β* usage within epitope-specific populations presents multiple challenges to frequency-based methods that have not been addressed to date, to our knowledge. The combination of alphabetr and relatively low-cost sequencing strategies addresses these issues, being capable of handling a wide range of clonal structures—skewed abundances, dual TCR*α*, sharing of both TCR*α* and TCR*β* between clones—as well as providing estimates of clonal abundances. The algorithm is available as a documented package in *R* [44] from http://github.com/edwardslee/alphabetr.

Single-cell technologies clearly allow the identification of large clonal expansions within populations. Our algorithm offers the potential to both identify these common clones as well as achieve depths of coverage of rarer clones that far exceed those currently possible with reasonable levels of single-cell sequencing. Given the correlation between diversity of immune responses and protection, this characterisation of the full diversity of T cell responses may be a better prognostic indicator than simply identifying common clones. Further, establishing the levels of TCR*α*- and TCR*β*-sharing within populations sheds light on mechanisms of antigen recognition, repertoire diversity, and the efficiency of recruitment into immune responses.

Our analysis demonstrates that the most difficult of these challenges is to reliably distinguish between abundant TCR*β*-sharing or dual TCR*α* clones within highly skewed populations because the expected patterns of co-occurrences of the three chains under the two alternatives are very similar when sequencing samples of a few tens of cells per well; all three chains typically appear in nearly all the wells. The difference in patterns can be magnified to an extent by sampling very few numbers of cells per well, but this solution comes with the cost of a reduction in total sample size, sacrificing depth of recovery of rarer clones. One might suppose that the high prevalence of dual TCR*α* clones in the naive T cell pool favours that scenario over TCR*β*-sharing. However, our immunological intuition here may be misleading. Naive T cell precursor numbers may be in the range 10–1000 cells in mice [45–47], which we estimate is comparable to or larger than the size of TCR*β*-sharing populations exported from the thymus. If the sharing of a TCR*β* between clones confers overlap in their TCR specificities, and if recruitment into immune responses is efficient, we might expect to see significant levels of TCR*β*-sharing within expanded, epitope-specific populations. Indeed, as shown in Table 1, TCR*β*-sharing has been seen to reach levels of up to 25% in responses to influenza epitopes in naive mice [13, 14] and almost 40% in secondary responses [14]. It also occurred at a level of 2% in our analysis of TCR*α* and TCR*β* usage among CD8^+^ cells specific for a YFV epitope in a human volunteer. The TCR*β*-sharing/dual TCR*α* ambiguity is therefore a robust feature of epitope-specific responses, and is challenging to unravel fully with statistical approaches.

There are at least three ways to address this problem. One solution is to pair alphabetr with, for example, one plate of single-cell samples. Since the ambiguity is only manifest strongly with common clones, this limited amount of extra information may serve to resolve the issue. A second approach is to exploit the fact that 30%-40% of clones will yield both an in-frame and an out-of-frame CDR3*α* sequence [13]. Currently, out-of-frame sequences are not utilised by alphabetr; one could extend it to include them and associate clones with their out-of-frame sequences. Clones possessing one in-frame and one out-of-frame CDR3*α* could then be excluded from the list of dual TCR*α* candidates, which would assist *β*-sharing/dual TCR*α* discrimination. A third possibility is to extend the algorithm to exploit the sequence information itself. If dealing with epitope-specific populations, we might expect more sequence similarity in the CDR3*α* in two *β*-sharing clones than in a dual TCR*α* case. In the latter, the two CDR3*α* sequences are likely unrelated because presumably only one of the TCR*α* chains is involved in antigen recognition and they rearrange independently.

In practice, one needs a strategy for implementing alphabetr on a given sample of T cells with no *a priori* knowledge of the number or size distribution of clones. Assuming the number of cells is not limiting, we advocate a high-mixed sampling approach that involves sampling 20–300 cells per well and deals efficiently with a wide range of clonal abundances. When alphabetr is implemented as described here, a standard desktop computer with 16 Gb of RAM is able to handle samples from parent distributions of up to 4000 clones. When sampling populations with much fewer clones, lower numbers of cells/well are needed to avoid high false pairing rates. Assuming cell numbers are not limiting, bulk sequencing of the *β* chain could be used to gain a rough estimate of the richness of the parent distribution and so indicate when a sparse sampling strategy would be beneficial. In situations where cell numbers are limiting, one approach could be to begin with a single plate of 10 cells/well to obtain a rough lower bound on the richness of the distribution and apply a low or high mixed sampling strategy with the remaining cells from the sample, as appropriate. The single plate of 10 cells/well is then still usable for the pairing process and for frequency estimation.

While we have framed our analysis around the sequencing of epitope-specific populations, alphabetr can equally well be applied more generally to T cell populations of restricted and potentially skewed polyclonality, such as tumour infiltrating lymphocytes or T cells extracted from sites of autoimmune responses. It therefore has immediate applications in cancer immunotherapy and other personalised immunomodulatory treatments. Until single-cell sequencing becomes more affordable, frequency-based pairing methods provide a rapid and economical means of characterising the clonal structure of T cell populations.

## Methods

### Ethics statement

All experimental procedures were approved by the Regional Ethical Review Board in Stockholm, Sweden: 2008/1881-31/4, 2013/216-32, and 2014/1890-32.

### Algorithm for TCR*αβ* pairing

Our approach exploits the fact that TCR*α* and TCR*β* sequences (referred to as *α* and *β* chains) will tend to appear together in wells. Let *N*_*α*_ be the total number of unique *α* chains, *N*_*β*_ be the total number of unique *β* chains, and the *α* and *β* chains found in the data set be labelled from 1 to *N*_*α*_ and from 1 to *N*_*β*_ respectively. The degree of association between chains *α*_*i*_ and *β*_*j*_ is measured by a score *S*_*ij*_,

$$S_{ij}=\sum_{k=1}^{W}\left(\frac{\delta_{ij}^{k}}{c_{\alpha }^{k}}+\frac{\delta_{ij}^{k}}{c_{\beta }^{k}}\right),$$ (1)

where the wells in the data are labelled from 1, 2, …, *W*, the numbers of distinct *α* and *β* chains in well *k* are $c_{\alpha }^{k}$ and $c_{\beta }^{k}$ respectively, and $\delta_{ij}^{k}$ is 1 if both *α*_*i*_ and *β*_*j*_ are found in well *k* and 0 otherwise. Eq 1 sums the co-appearances in wells, each weighted inversely by the total number of *α* and *β* chains recovered from the well. The scaling accounts for the fact that the larger the number of unique chains in a well, the lower our confidence that a co-occurring *α* and *β* pair derive from the same clone.

The algorithm begins by sampling a proportion *p*_*J*_ of the wells in the data without replacement. For all analyses presented here, we used *p*_*J*_ = 0.75, which provided a good balance between depth and false pairing rate. The algorithm computes the association scores between every unique *α* and *β* chain using Eq 1 based on the sampled subset of wells. Let $A_{k}$ denote the set of *A* distinct *α* chains found in well *k*, that is $A_{k}=\{\alpha_{m_{1}^{k}},\alpha_{m_{2}^{k}},\ldots ,\alpha_{m_{A}^{k}}\}$, where the $m_{i}^{k}\in \left\{1,\ldots ,N_{\alpha }\right\}$ are integers that denote the labels of the *A* TCR*α* chains found in well *k*. Similarly, let $B_{k}$ denote the set of *B* distinct *β* chains found in well *k*, that is $B_{k}=\{\beta_{n_{1}^{k}},\beta_{n_{2}^{k}},\ldots ,\beta_{n_{B}^{k}}\}$, where the $n_{i}^{k}\in \left\{1,\ldots ,N_{\beta }\right\}$ subscripts denote the labels of the *B* TCR*β* chains found in well *k*. The algorithm solves the following linear assignment problem using the Hungarian algorithm [39]:

$$\begin{array}{ll} \text{maximize} & \sum_{\alpha_{i}\in A_{k}}\sum_{\beta_{j}\in B_{k}}S_{ij}x_{ij}\\ \text{subject to} & \sum_{\alpha_{i}\in A_{k}}x_{ij}=1\text{ for }\beta_{j}\in B_{k}\\ & \sum_{\beta_{j}\in B_{k}}x_{ij}=1\text{ for }\alpha_{i}\in A_{k}\\ & x_{ij}\geq 0,\;\alpha_{i}\in B_{k},\;\beta_{j}\in A_{k}, \end{array}$$ (2)

where *x*_*ij*_ = 1 indicates that *α*_*i*_ and *β*_*j*_ are assigned as a candidate TCR pair and *x*_*ij*_ = 0 otherwise. A pair *α*_*i*_*β*_*j*_ is defined as an assigned pair of well *k* if *x*_*ij*_ = 1 for Eq 2 associated with well *k*. The number of assignments made for every pair of *α* and *β* is recorded as *X*_*ij*_, i.e. *X*_*ij*_ equals the number of times *x*_*ij*_ = 1 from the solutions of Eq 2 for each well in the subset. We then calculate a filter level *F* that determines the minimum number of assignments required for an assigned candidate pair of *α* and *β* chains to be determined as a true TCR pair. The filter-level *F* is chosen to be the mean of the elements of the set {*N*(*i*, *j*) : *N*(*i*, *j*) > 0, *i* ∈ 1, 2, …, *N*_*α*_, *j* ∈ 1, 2, …, *N*_*β*_}, where *N*(*i*, *j*) is the number of times *α*_*i*_ *β*_*j*_ are assigned to each other, The output of this algorithm is then a list of candidate *αβ* pairs that may be associated with T cell clone. At this stage, dual TCR*α* cells are not identified; thus a clone *α*_1_*α*_2_*β* may be represented in this list as one or both of *α*_1_*β* and *α*_2_*β*.

The procedure above is performed *N*_*r*_ times on random subsets of the wells (all simulations in this paper use *N*_*r*_ = 100), and each replicate yields a list of candidate *αβ* pairs. We then perform a filtering or consensus step in which only *αβ* pairings that appear in more than a threshold proportion *T* of these lists are retained as candidates. The simulations we present in the text explore thresholds of *T* = 0.3, 0.6, and 0.9.

### Maximum-likelihood estimation of clonal frequencies

We use maximum likelihood to infer clonal frequencies based on the number of wells in which a pair of *α* and *β* chains both appear. Let *N* = {*n*_1_, *n*_2_, …, *n*_*s*_} be the set of *s* distinct sample sizes (*n*_*i*_ cells per well) in all of the wells and *W* = {*w*_1_, *w*_2_, …, *w*_*s*_} where *w*_*i*_ represents the number of wells with samples of size *n*_*i*_ cells. Let *c*_*ij*_ denote the clone with chains *α*_*i*_ and *β*_*j*_ and let $k_{ij}^{l}$ denote the number of wells of sample size *n*_*l*_ cells per well that contain chains *α*_*i*_ and *β*_*j*_. The likelihood of the observations $k_{ij}^{\left(.\right)}$, given that the clone *c*_*ij*_ is present at frequency *f*_*ij*_ within the population, is

$$L\left(\text{observed}\;\text{incidence}\;\text{of}\;\text{clone}\;c_{ij}\;|\;f_{ij}\right)=\prod_{l=1}^{s}\left(\begin{array}{c} w_{l}\\ k_{ij}^{l} \end{array}\right){\left(1-q_{l}\right)}^{k_{ij}^{l}}q_{l}^{w_{l}-k_{ij}^{l}}$$ (3)

where *q*_*l*_ is the probability of clone *c*_*ij*_ not being found in well *l* and is given by

$$q_{l}={\left(1-f_{ij}\right)}^{n_{l}}+\sum_{m=1}^{n_{l}}\left(2\epsilon^{m}-\epsilon^{2m}\right)\left(\begin{array}{c} n_{l}\\ m \end{array}\right)f_{ij}^{m}{\left(1-f_{ij}\right)}^{n_{l}-m}.$$ (4)

Here *ϵ* is the average probability that a CDR3 sequence in a cell fails to be amplified and sequenced. For every clone *c*_*ij*_, the algorithm maximises Eq 3 to estimate its frequency *f*_*ij*_, and 95% confidence intervals are defined by the frequencies yielding $\log L=\log L_{\text{max}}-\text{1.96}$. Details of the derivation of Eqs 3 and 4 are given in Section 4 of S1 Text.

This procedure is applied to every *αβ* pair identified in the first phase of the algorithm. These estimated frequencies are used to distinguish TCR*β*-sharing clone pairs from single TCR clones expressing two TCR*α*. This procedure is described in the following section. When a clone with two TCR*α* is identified, we revise the frequency estimate as follows. Let *c*_(*ij*)*t*_ denote a clone with chains *α*_*i*_, *α*_*j*_, and *β*_*t*_, and $k_{(ij)t}^{l}$ denote the number of wells of size *n*_*l*_ that contain chains *α*_*i*_, *α*_*j*_, and *β*_*t*_. The likelihood of the observations given that clone *c*_(*ij*)*t*_ has a frequency *f*_(*ij*)*t*_ ∈ (0, 1] is

$$L\left(\text{observed}\;\text{incidence}\;\text{of}\;\text{clone}\;c_{(ij)t}\;|\;f_{(ij)t}\right)=\prod_{l=1}^{s}\left(\begin{array}{c} w_{l}\\ k_{(ij)t}^{l} \end{array}\right){\left(1-q_{l}\right)}^{k_{(ij)t}^{l}}q_{l}^{w_{l}-k_{(ij)t}^{l}}$$ (5)

where *q*_*l*_ is the probability of clone *c*_(*ij*)*t*_ not being found in well *l* and is given by

$$q_{l}={\left(1-f_{(ij)t}\right)}^{n_{l}}+\sum_{m=1}^{n_{l}}\left(3\epsilon^{m}-3\epsilon^{2m}+\epsilon^{3m}\right)\left(\begin{array}{c} n_{l}\\ m \end{array}\right)f_{(ij)t}^{m}{\left(1-f_{(ij)t}\right)}^{n_{l}-m}$$ (6)

where *ϵ* is the mean drop rate as described above. Eq 5 is then maximised to estimate *f*_(*ij*)*t*_, and again $\log L=\log L_{\text{max}}-\text{1.96}$ is used to calculate 95% confidence intervals.

### Discriminating between dual TCR*α* and shared TCR*α* chains

If the algorithm yields two clones that appear to share a TCR*β* (*α*_1_*β* and *α*_2_*β*), we must decide whether this is indeed a *β*-sharing pair of clones or that the association derives from one dual TCR*α* clone (*α*_1_*α*_2_*β*). To do this, we use the likelihoods of observed co-occurrences of the three chains to assess the relative support for the two alternatives.

Let *c*_*ij*_ = (*α*_*i*_, *β*_*j*_) and *c*_*kj*_ = (*α*_*k*_, *β*_*j*_) be two putative clones with a common TCR*β* chain *β*_*j*_. We count the number of wells containing all three-way, two-way, and single appearances of the three chains. We then calculate the ‘full’ likelihoods of this pattern of occurrences under two hypotheses: (A) that *c*_*ij*_ and *c*_*kj*_ are indeed two *β*-sharing clones, with frequencies *f*_*ij*_ and *f*_*kj*_ estimated using Eq 3; and (B) that the chains derive from one dual TCR*α* clone *c*_(*ij*)*k*_ present at frequency *f*_(*ij*)*k*_, estimated using Eq 5. If the difference $\log L_{B}-\log L_{A}\geq 10$, we assume the three chains derive from dual TCR*α* clone.

The calculation of these full likelihoods is in Section 6 of S1 Text but is computationally tractable only for wells with less than 50 cells due to the need to calculate large multinomial coefficients. The full-likelihood method is therefore only appropriate for estimating frequencies of those relatively abundant clones that are commonly found in the wells with smaller sample sizes. We use a more restricted likelihood-based approach for discriminating *β*-sharing and dual TCR*α* among rare clones, which tend to appear only in larger samples. Let clones *c*_*ij*_ = (*α*_*i*_, *β*_*j*_) and *c*_*kj*_ = (*α*_*k*_, *β*_*j*_) be two clones with a common beta chain *β*_*j*_, and let *f*_*ij*_ and *f*_*kj*_ be their estimated frequencies. The algorithm calculates the ratio $r_{ik}^{j}$ of the observed to the expected number of wells in which all three chains from the putative *β*-sharing pair *c*_*ij*_ and *c*_*kj*_ co-appear, under the hypothesis that they are indeed two clones and not a dual TCR*α*:

$$R=\left\{r_{ik}^{j}=\frac{A\left(c_{ij},c_{kj}\right)}{E\left(c_{ij},c_{kj}\right)}:i\neq k,\;j\in 1,2,\ldots ,N_{\beta }\right\}$$ (7)

where *A*(*c*_*ij*_, *c*_*kj*_) is the number of times clones *c*_*ij*_ and *c*_*kj*_ are observed to appear in the same well and *N*_*β*_ is the number of distinct *β* chains, and the expected number is

$$E\left(c_{ij},c_{kj}\right)=\sum_{l=1}^{s}w_{l}\left(1-{\left(1-f_{ij}\right)}^{n_{l}}-{\left(1-f_{kj}\right)}^{n_{l}}+{\left(1-f_{ij}-f_{kj}\right)}^{n_{l}}\right)$$ (8)

(see S1 Text, Section 5 for a derivation and discussion of this equation). We then partition the set of ratios *R* into two groups *C*_1_ and *C*_2_ using *k*-means clustering, where the mean of ratios of *C*_1_ is greater than the mean of the ratios of *C*_2_ (see S1 Text, Fig G for an example). The clones associated with the ratios in *C*_1_ are chosen as dual TCR clones, such that if $r_{ik}^{j}\in C_{1}$, then clones *c*_*ij*_ and *c*_*kj*_ are removed from the list of TCR pairs and replaced with a dual TCR*α* clone *α*_*i*_ *α*_*k*_ *β*_*j*_.

### Creation of *in silico* data sets for validation

We created synthetic data sets reflecting the properties of antigen-specific T cell populations and sequencing errors. The data sets were sampled from a population of T cell clones where a significant proportion of *α* and *β* chains are shared and 10%-30% of clones have dual TCR*α* chains (e.g. three clones can have the following chains: *α*_*i*_ *β*_*k*_, *α*_*j*_ *β*_*k*_, and *α*_*j*_ *α*_*h*_ *β*_*l*_). The sharing of *β* chains was set such that 85.9% of *β* chains were uniquely from one clone, 7.6% shared by two clones, 3.7% shared by three clones, 1.9% by four clones, and 0.9% by five clones. The sharing of *α* chains was set such that 81.6% of *α* chains were uniquely from one clone, 8.5% shared by two clones, 2.1% shared by three clones, 0.7% shared by four clones, 3.3% shared by five clones, 0.5% shared by six clones, and 3.3% shared by seven clones. We determined these levels of sharing by averaging those from the published single-cell data shown in Table 1.

The frequencies of the *N* clones were drawn from a skewed distribution in which *n*_*s*_ clones comprise a proportion *p*_*s*_ of the population and the other *N* − *n*_*s*_ clones evenly represent 1 − *p*_*s*_ of the population. The clone ranked *i*^th^ in abundance then has frequency *f*_*i*_ where

$$f_{i}=\left\{\begin{array}{cc} f_{1}+r\left(i-1\right) & \text{if}\;i=1,\;2,\;\ldots ,\;n_{s}\\ p_{s}/\left(N-n_{s}\right) & \text{if}\;i=n_{s}+1,\;n_{s}+2,\;\ldots ,\;N \end{array}\right.$$ (9)

where the frequency of the largest clone *f*_1_ and the step size *r* are determined by solving the equations

$$\sum_{i=1}^{n_{s}}f_{i}=p_{s},\;f_{n_{s}}=1.1\times \frac{p_{s}}{N-n_{s}}.$$ (10)

The frequency of the smallest clone in the top 50%, *f*_*n*_*s*__, is set to be 10% higher than the frequency of the clones in the tail. All simulations were based on *p*_*s*_ = 0.5. We varied the number of top clones *n*_*s*_ between 5 to 50 to test how skewness in the antigen-specific T cell population impacts the performance of the algorithm.

In order to make the simulated data more realistic, experimental noise was included in the forms of ‘dropped’ chain errors and in-frame sequencing errors. Dropped chains are CDR3 sequences that fail to be sequenced due to PCR errors and/or sorting problems, and studies utilising both single-cell and many-cell techniques have reported average drop rates of 8% to 10% [17, 22]. In the simulations, each clone was assigned a drop rate from a lognormal distribution with a mean of 0.15 and standard deviation of 0.01, and every TCR*α* and TCR*β* chain belonging to that clone was assigned that drop rate. In-frame errors cause a CDR3 sequence to be falsely identified with an incorrect productive nucleotide and/or amino acid sequence. In the simulations, each distinct sequence was assigned an in-frame error rate drawn from a lognormal distribution with a mean of 0.02 and a standard deviation of 0.005. The error model was simulated as follows: when a cell is sampled into a virtual well, each of its chains fails to be sequenced with probability equal to the pre-assigned, clone-specific drop rate. Every surviving chain produces one of three randomly chosen, distinct, and chain-specific false sequences with probability equal to that chain’s pre-assigned in-frame error rate.

### TCR sequencing

A human volunteer was identified as HLA-A2^+^/HLA-B7^+^ and received the live attenuated yellow fever vaccine (YFV-17D). On day 15 post-vaccination, peripheral blood samples were taken, and live CD3^+^CD8^+^ T cells were isolated by negative selection using magnetic columns (Miltenyi Biotec, CD8^+^ T cell negative isolation kit). Cells were labeled with a panel of antibodies and the HLA-A02:01/LLWNGPMAV dextramer representing the immunodominant response. Single dextramer-specific CD3^+^CD8^+^ T cells were sorted into individual wells in 96 well plates containing a lysis buffer (0.4% Triton, RNAse inhibitor, dNTP, OligodT) and immediately stored on dry ice. Single cell transcriptome libraries were subsequently generated from these cells using an adapted version of the SMRT-Seq2 protocol [48]. Libraries were prepared for sequencing by tagmentation and labelling individual single cell transcriptomes with a custom Tn5 enzyme [49] and Nextera XT dual indexes. Pooled libraries were then sequenced using an Illumina Hiseq2500 on high output mode (2 × 100bp or 2 × 125bp reads), and individual TCR*α* and TCR*β* chains were identified using the MiTCR algorithm with default parameters. The default settings for MiTCR were used to align the CDR3 sequences. These were then manually filtered to remove erroneous sequences (e.g. early stop codons and CDR3 sequences that were greater than 30 amino aids in length), and then BLAST was used on the remaining sequences to check for mapping to other parts of the genome, removing as appropriate. All clones used in the comparative analysis of CDR3*α* lengths were curated manually to exclude the possibility of contaminating TCR sequences.

CDR3 amino acid sequences are provided as a CSV file in S1 Dataset, and the raw reads are deposited in the Gene Expression Omnibus (GEO), GSE75659; Sequence Read Archive (SRA), SRP066963.

## Supporting Information

S1 Text Supporting analyses. (PDF)

S1 Dataset TCR sequences of HLA-A02:01/LLWNGPMAV-specific cells. (CSV)
