# Supplementary material for: Identifying T Cell Receptors from High-Throughput Sequencing: Dealing with Promiscuity in TCRα and TCRβ Pairing
Source: PLoS Comput Biol. 2017 Jan 19;13(1):e1005313. doi: 10.1371/journal.pcbi.1005313 (PMC5289640; doi:10.1371/journal.pcbi.1005313)
Supplement: S1 Text — (PDF) [file pcbi.1005313.s001.pdf]

S1 Text – Supporting Information

1 Nearly all TCRβ chains recovered from typically-sized samples of human naive $\alpha\beta$ T cells are expected to be unique

Let B be the number of unique β chains found in the naive $\alpha\beta$ T cell repertoire. How many unique β chains U_β we would expect to find in a sample of N naive $\alpha\beta$ T cells from human blood, assuming $N \ll 10^{11}$, the total naive T cell population size? This problem is equivalent to randomly sampling with replacement N times from a set of B different β chains labelled $\beta_1, \beta_2, \ldots, \beta_B$ and asking how many of them appear in that sample on average. This is straightforward to calculate if one assumes that each distinct β chain is present roughly at equal frequency within the naive T cell pool. For any non-uniform distribution of naive TCRβ clone sizes [1, 2], the following calculation provides a lower bound on the sample size required to achieve a given level of coverage of the repertoire.

Let I_i be a random variable equal to 1 if chain β_i appears in the sample at least once and 0 otherwise. $P(I_i = 0)$ is $((B-1)/B)^N$, and so its expected value $\langle I_i \rangle$ is $1 - ((B-1)/B)^N$. The expected number of different β chains in the sample is then

$$U_\beta = \left\langle \sum_{i=1}^{B} I_i \right\rangle = \sum_{i=1}^{B} \langle I_i \rangle = B\left(1 - \left(\frac{B-1}{B}\right)^N\right). \tag{1.1}$$

Similarly, let J_i be a random variable that is equal to 1 if clone i appears only once in the sample, and zero otherwise. $P(J_i = 1)$ is $(N/B)(1-1/B)^{N-1}$, and so the expected number of chains that appear only once is $B \cdot \langle J_i \rangle = N(1-1/B)^{N-1}$. Figure A shows the dependence of these quantities on sample size using the current best estimate of B in humans, 10^8 [3].

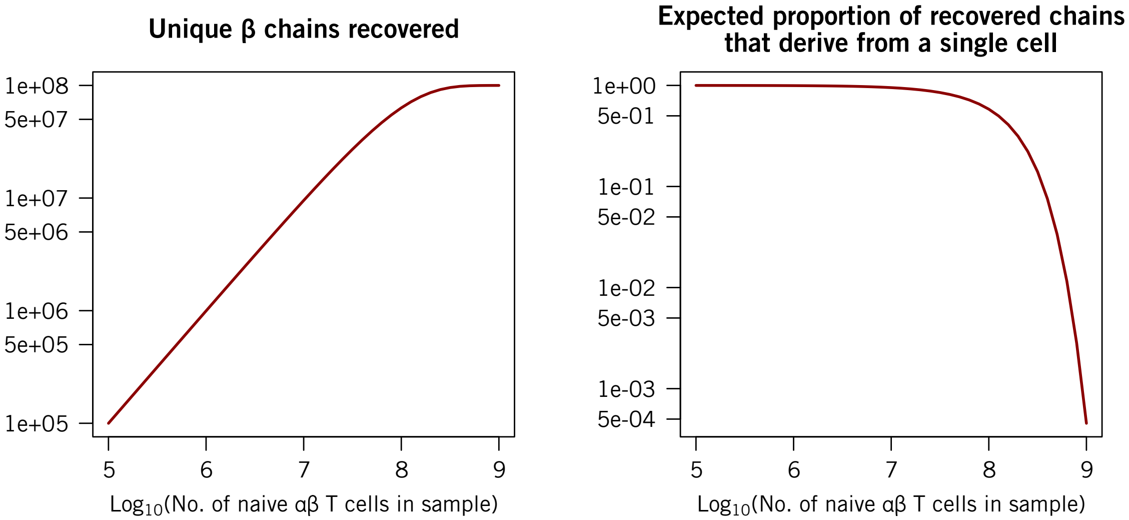

Figure A: Expected number of unique β chains recovered from a large population with a repertoire of 10^8 different β chains, for varying sample sizes (sampling with replacement).

We can then estimate typical TCRβ clone size distributions obtained from bulk sequencing of T cells recovered from human blood samples. One millilitre of human blood yields typically 0.5×10^6 to 1.8×10^6 $\alpha\beta$ T cells, of which 0.1×10^6 to 0.8×10^6 are naive CD4 T cells and 0.03×10^6 to 0.2×10^6 are naive CD8 T cells. A 10 mL blood sample will then contain between 10^6 and 10^7 naive $\alpha\beta$ T cells. Eq 1.1 predicts that this will yield 1-10% of the β chain repertoire, with correspondingly 99.5% and 95% of the chains recovered deriving from a single cell. In a 50 mL sample, we expect to cover between 5% and 40% of the β chain repertoire, with correspondingly 98% and 80% of the chains deriving from only one cell.

2 Evaluation of the performance of ALPHABETR under different experimental conditions

In order to evaluate the robustness of ALPHABETR, we performed simulations to measure its ability to determine TCR pairs from a wide range of antigen-specific T cell populations with different levels of sharing, with different numbers of distinct clones, and without the presence of dual TCRβ chains.

Figures B-C show the results of simulations of populations with higher and lower levels of TCRα- and TCRβ-sharing respectively. All other parameters and error models were identical to those in the main text. The higher sharing simulations in Figure B assumed 20% of TCRα and TCRβ chains shared by two clones, 10% of TCRα and TCRβ shared by three clones, and 10% of TCRα and TCRβ shared by four clones (a total of 40% of all distinct chains being shared by at least two clones). The lower sharing simulations in Figure C included 5% of TCRα and TCRβ chains shared by two different clones and 5% of TCRα and TCRβ shared by three clones (a total of 10% of all distinct chains being shared by at least two clones). Higher sharing levels have a minimal effect on top and tail depths while causing an absolute increase in false pairing rates of only 1%-2%. Lower sharing levels also appear to have a minimal effect on top and tail depth while causing an absolute decrease in false pairing rates of approximately 1%.

Figures D-E show the results of simulations of populations comprising 3000 and 500 clones respectively to assess the effect of increased and decreased diversity on the performance of ALPHABETR. Error models and levels of sharing were as those used in the main text. Figure D shows that ALPHABETR maintains the same top depth for more diverse populations while obtaining slightly lower tail depths, which is not surprising given that larger total sample sizes are needed to achieve coverage of the larger tail of more diverse populations. There is no substantial difference in false pairing rates. In Figure E, the simulations show that ALPHABETR has similar top depths and higher tail depths for low-diversity populations. The higher false pairing rates seen here are due to the fact that using both of the mixed sampling strategies described in the main text (Table 2) involve sufficiently large numbers of cells per well. For low-diversity populations, common clones will appear together in wells very often with these sample sizes, creating ambiguity in pairing and increasing the apparent degree of sharing of chains between clones.

Figure F shows the results of simulations of populations with no dual-TCRβ clones since these have been rarely reported in the literature. All other aspects of the simulated data are the same as those reported in the main text. For 5 plates and a moderate consensus threshold of 0.6, the simulations show up to 98% recovery of common clones and 61% recovery of rare clones with false pairing rates of less than 3.4%. The false pairing rate falls below 1% at a stringent threshold of 0.9.

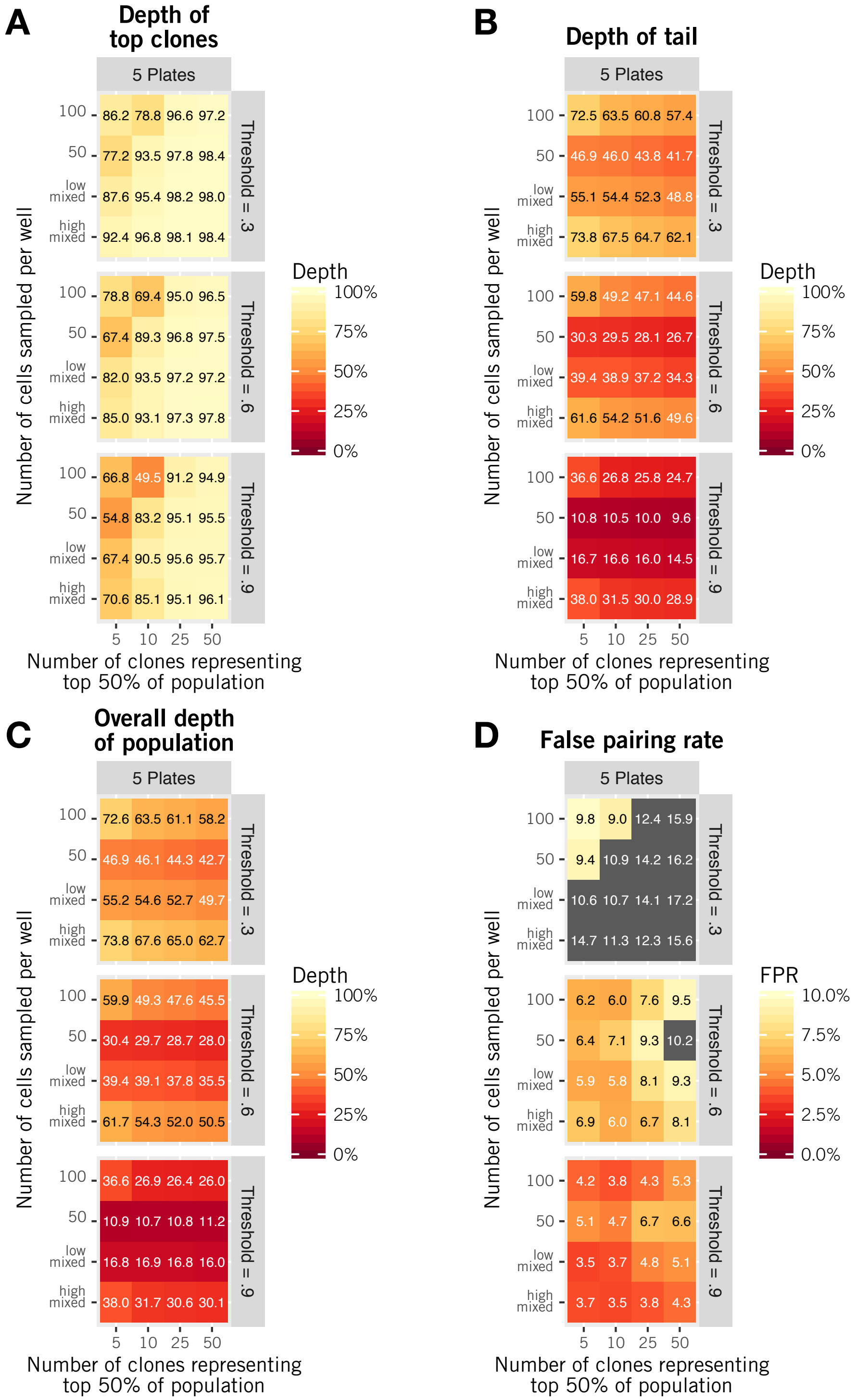

Figure B: **High sharing.** Performance of ALPHABETR at a high level of chain sharing with 20% of TCRα and TCRβ chains shared by two different clones, 10% of TCRα and TCRβ shared by three clones, and 10% of TCRα and TCRβ shared by four clones. The results shown are the averages of 100 simulations.

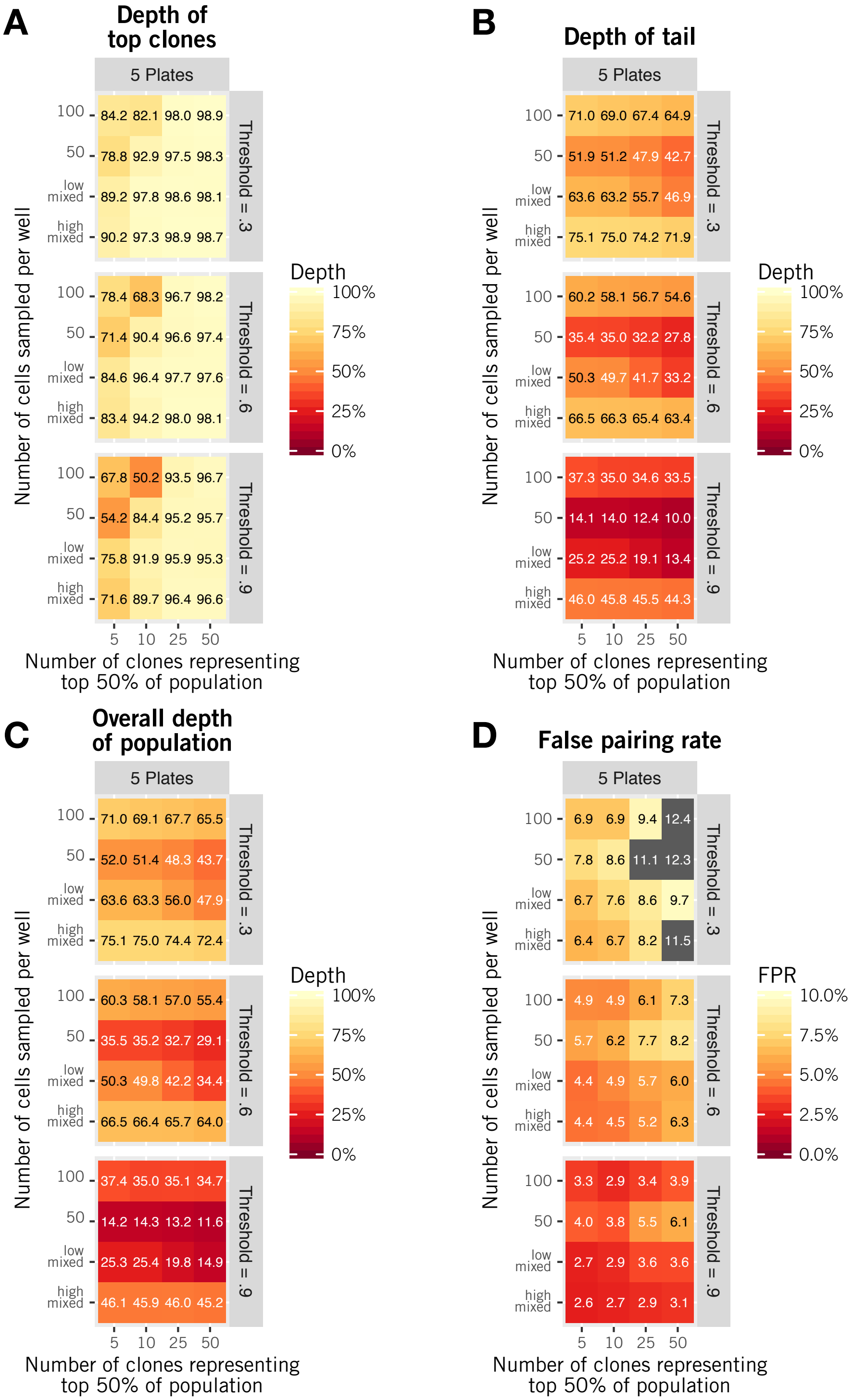

Figure C: **Low sharing.** Performance of ALPHABETR at low level of chain sharing with 5% of TCRα and TCRβ chains shared by two different clones and 5% of TCRα and TCRβ shared by three clones. The results shown are the averages of 100 simulations.

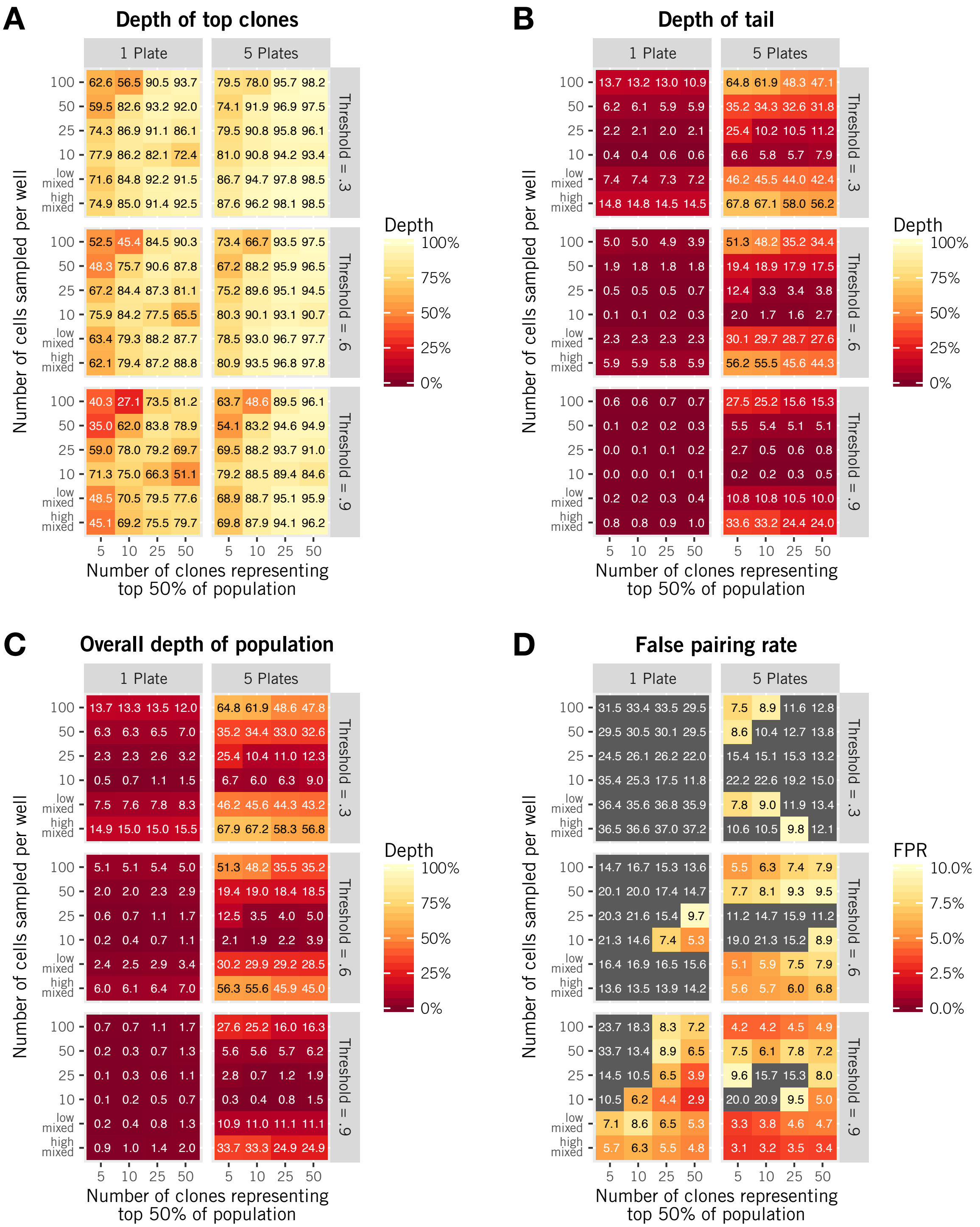

Figure D: **3000 clones.** Performance of ALPHABETR with 3000 clones in the parent T cell population. The results shown are the averages of 100 simulations.

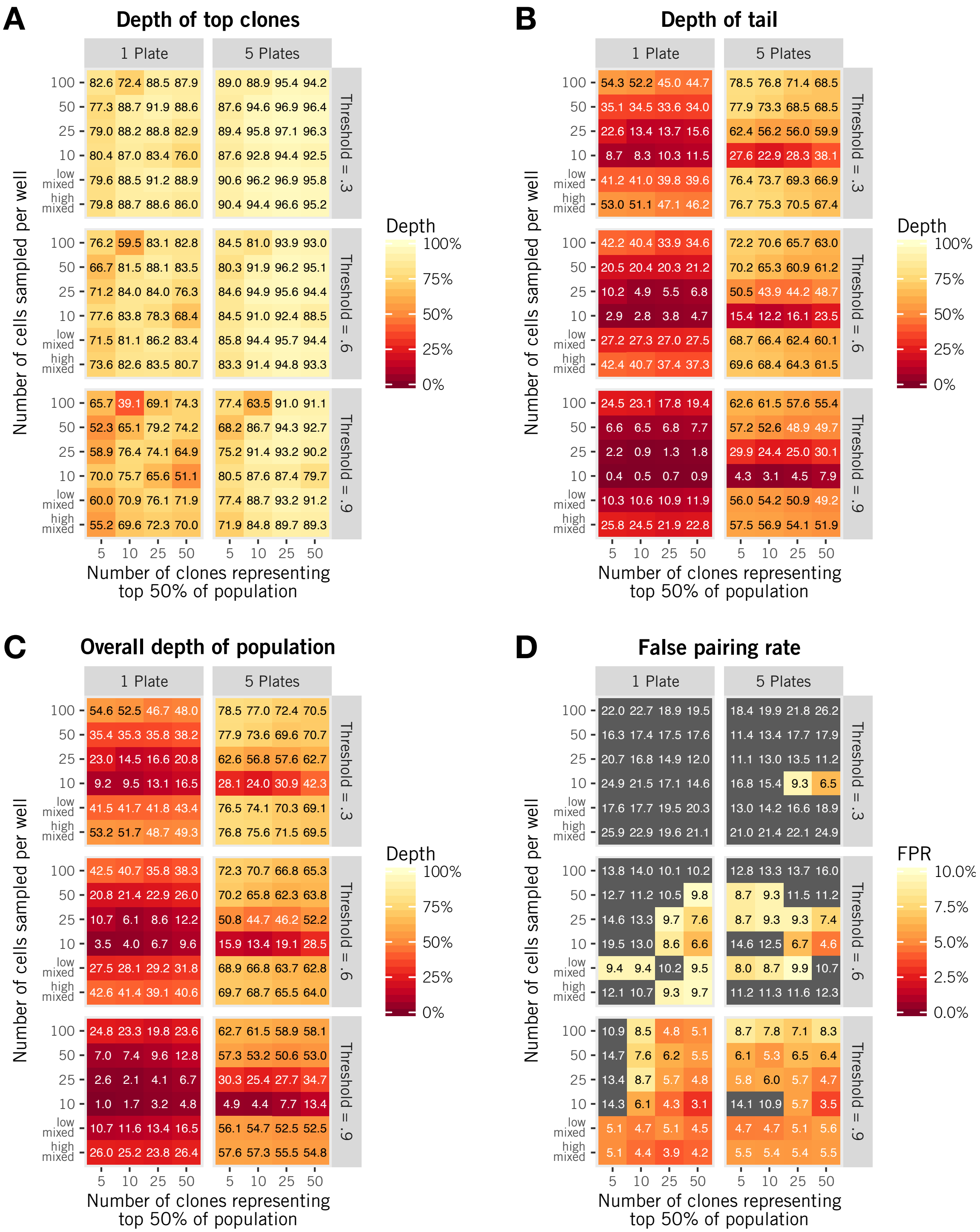

Figure E: **500 clones.** Performance of ALPHABETR with 500 clones in the parent T cell population. The results shown are the averages of 100 simulations.

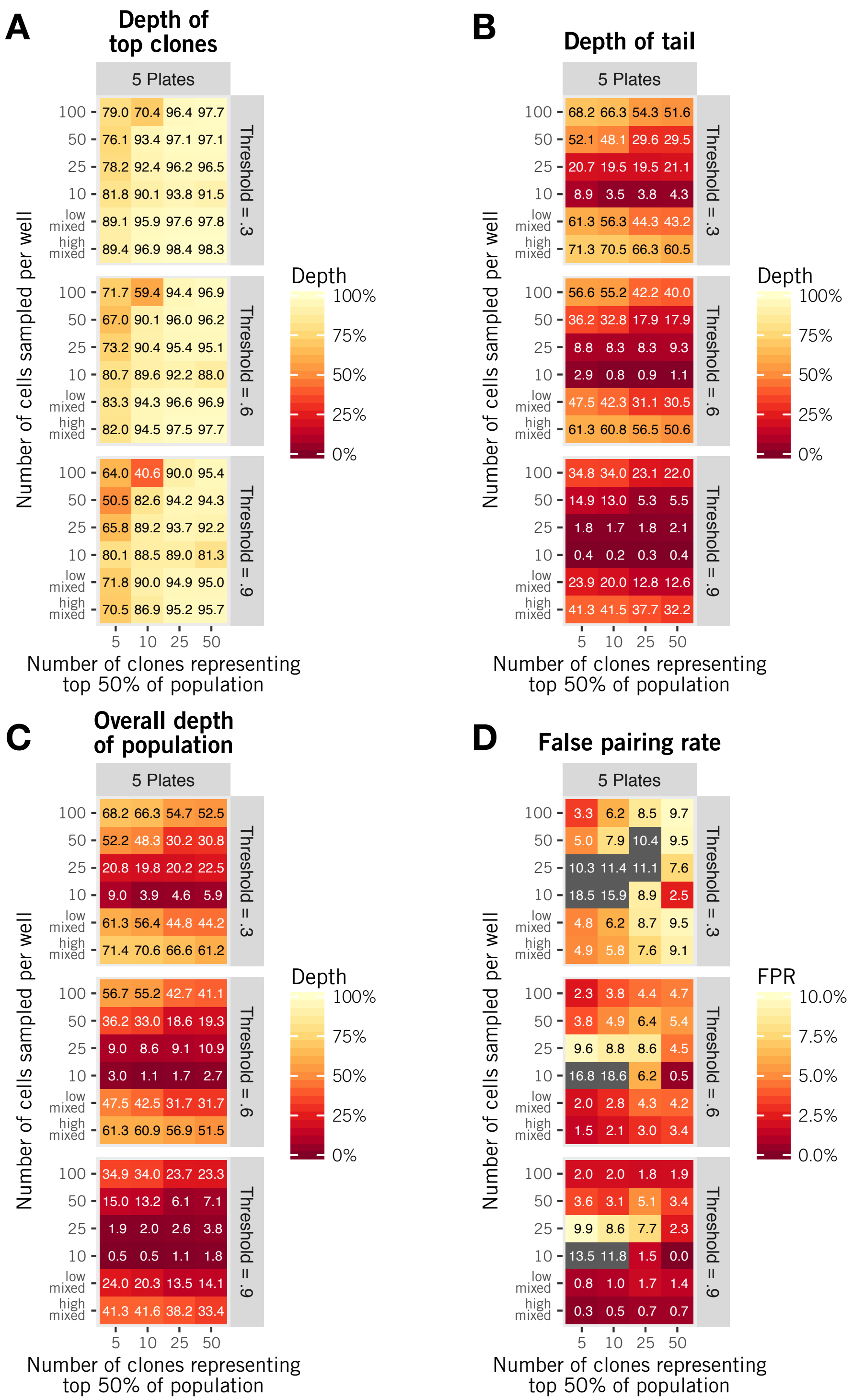

Figure F: **No dual-TCRβ clones.** Performance of ALPHABETR without dual β chains. The results shown are the averages of 100 simulations.

3 Accuracy and precision of clonal abundance estimates in the absence of information regarding the probability that CDR3 regions fail to be sequenced

We performed simulations to determine the sensitivity of clonal frequency estimation to inaccuracies in estimates of the average drop rate of CDR3α and CDR3β sequences. These simulations involved creating data sets of 5 plates, the high-mixed sampling strategy and a mean drop rate of $\epsilon = 0.15$. We then performed frequency estimation on these data sets, as described in Methods Section M2, using mean drop rates of $\epsilon = 0.15$, $\epsilon = 0.08$, and $\epsilon = 0$. We show the mean bias in these frequency estimates, where

$$\text{Bias} = \frac{\text{Estimated frequency} - \text{True frequency}}{\text{True frequency}}$$

Table A shows the results of 200 simulations for each combination of ϵ and skewness of the clone size distribution (number of clones in the top 50% of the population when ranked by clonal abundance). Assuming 100% efficiency of sequencing leads to underestimation of clonal frequencies. Bias in estimates also naturally leads to inaccuracy in the construction of 95% confidence intervals.

Error term input	Number of top clones	Percent of 95%-CI containing true frequency	Mean bias
ϵ = 0.15	5	91.5%	3.8%
	10	93.4%	2.4%
	25	92.7%	2.1%
	50	92.3%	3.0%
ϵ = 0.08	5	65.3%	-12.1%
	10	53.4%	-12.7%
	25	47.7%	-12.8%
	50	47.8%	-12.7%
ϵ = 0	5	31.2%	-25.2%
	10	12.8%	-26.4%
	25	6.7%	-26.3%
	50	7.8%	-26.2%

Table A: **Assessing the impact of underestimation of sequencing error on clonal frequency estimation.** Using accurate estimates of the drop rate (here, $\epsilon = 0.15$) results in accurate frequency estimates. Underestimating the drop rate leads to biased estimates that are lower bounds on the true frequencies.

4 Details of clonal frequency estimation

The maximum likelihood approach for clonal frequency estimation involves modelling how a clone is sampled in the wells of the plates. Since we assume conservatively that sequencing does not give any quantitative information about the number of times a clone is sampled in a well, the data fed to ALPHABETR need to indicate only whether a given CDR3α or CDR3β sequence is present in each well. Let s denote the number of distinct sample sizes placed in the wells; $\mathcal{N} = \{n_1,\ n_2,\ \ldots, n_s\}$ be the set of s distinct sample sizes where n_i is number of cells per well; and $\mathcal{W} = \{w_1,\ w_2,\ \ldots, w_s\}$ be the set where w_i represents the number of wells with sample size n_i. Let c_{ij} denote the clone with chains α_i and β_j, and let k_{ij}^l denote the number of wells of size n_l cells per well that contain α_i and β_j.

The likelihood of clone $\alpha_i \beta_j$ appearing in k_{ij}^l wells of sample size n_l cells for $l = 1, \ldots, s$ is the probability of the clone being sampled in k_{ij}^l out of the w_l possible wells, which is

$$P(k_{ij}^l \text{ wells}|\text{ frequency } f_{ij}) = \binom{w_l}{k_{ij}^l} P(\text{clone sampled in well of size } n_l)^{k_{ij}^l} \times \\ \Big(1 - P(\text{clone sampled in well of size } n_l)\Big)^{w_l - k_{ij}^l} \tag{4.1}$$

We define $q_l = 1 - P(\text{clone sampled in well of size } n_l)$, which the probability of clone $\alpha_i \beta$ not being sampled in a well of size n_l. Since the k_{ij}^l appearances are independent, the probability of observing is determined by summing Eq (4.1) for all sample sizes and is given by

$$P(\text{observations} = k_{ij}^1, k_{ij}^2, \ldots, k_{ij}^s \text{ wells } | \text{ frequency } f_{ij}) = \prod_{l=1}^{s} \binom{w_l}{k_{ij}^l} (1 - q_l)^{k_{ij}^l} q_l^{w_l - k_{ij}^l}, \tag{4.2}$$

which is Eq (3) in the main text. The probability q_l is calculated by adding the probabilities of all of the events that would result in the clone not being sampled in the well, which are:

- A: clone $\alpha_i \beta_j$ not being sampled at all
- B: clone $\alpha_i \beta_j$ is sampled $m \leq n_l$ times (resulting in m copies of chains α_i and β_j), all m copies of α_i are dropped, and at least 1 copy of β_j is not dropped
- C: clone $\alpha_i \beta_j$ is sampled $m \leq n_l$ times, at least 1 copy of α_i chain is not dropped, and all m copies of β_j are dropped
- D: clone $\alpha_i \beta_j$ is sampled $m \leq n_l$ times, and all m copies of α_i and m copies of β_j are dropped.

The probability of event A is given by

$$P(A) = (1 - f_{ij})^{n_l} \tag{4.3}$$

Events B and C are symmetric, and the probability of these events is the probability that the clone will be sampled $m \leq n_l$ times multiplied by the probability of dropping all of one of the chains and not dropping at least one of the other chains. This is given by

$$P(B) = P(C) = \sum_{m=1}^{n_l} \binom{n_l}{m} (f_{ij})^m (1 - f_{ij})^{n_l - m} \epsilon^m (1 - \epsilon^m) \tag{4.4}$$

where ϵ^m is the probability of dropping m of one of the component chains. The probability of event D is derived similarly:

$$P(D) = \sum_{m=1}^{n_l} \binom{n_l}{m} (f_{ij})^m (1 - f_{ij})^{n_l - m} \epsilon^m \epsilon^m. \tag{4.5}$$

Summing these yields Eq (4) in Methods of the main text.

The likelihood for dual clones is obtained in a similar fashion, where q_l is calculated by summing the probabilities of the clone not being sampled at all and of being sampled but dropping one, two, or all three of the clone's chains.

5 Using k-means clustering to distinguish rare β-sharing and dual TCRα clones

The ratios in Eq (7) in Methods involve the expected number of wells in which the three chains $\alpha_1\alpha_2\beta$ co-occur under the assumption that they derive from two β-sharing clones $\alpha_1\beta$ and $\alpha_2\beta$. We derive that quantity here. Let c_{ij} and c_{kj} be two clones that share β_j. Let A^l_{ij} and A^l_{kj} denote the events of sampling clones c_{ij} and c_{kj} in a well of size n_l cells respectively and $A^{l^C}_{ij}$ and $A^{l^C}_{kj}$ denote the complement of these events. The probability of sampling both clones in a well of n_l cells is then

$$P(A^l_{ij} \cap A^l_{kj}) = 1 - P(A^{l^C}_{ij} \cup A^{l^C}_{kj}) \tag{5.1}$$

$$= 1 - \left(P(A^{l^C}_{ij}) + P(A^{l^C}_{kj}) - P(A^{l^C}_{ij} \cap A^{l^C}_{kj})\right) \tag{5.2}$$

In calculating Eq (5.2), including the effect of stochastic dropping of chains results in large multinomial coefficients that cannot be computed efficiently for wells with larger sample sizes (approximately ≥ 50 cells per well). Heuristically, however, neglecting the drop rate has no impact on discrimination of β-sharing and dual TCRα. We then have

$$P(A^{l^C}_{ij}) = (1 - f_{ij})^{n_l} \tag{5.3}$$

$$P(A^{l^C}_{ik}) = (1 - f_{kj})^{n_l} \tag{5.4}$$

$$P\left(A^{l^C}_{ij} \cap A^{l^C}_{kj}\right) = \left(1 - (f_{ij} + f_{kj})\right)^{n_l} \tag{5.5}$$

By substituting Eqs (5.3)-(5.5) into Eq (5.2) and multiplying by w_l (the total number of wells of size n_i), we obtain the expected number of wells of size n_i that contain both clones:

$$E(c_{ij}, c_{kj}) = w_l\left(1 - (1 - f_{ij})^{n_l} - (1 - f_{kj})^{n_l} + (1 - f_{ij} - f_{kj})^{n_l}\right). \tag{5.6}$$

By summing this quantity over all wells and sample sizes, we obtain Eq (8) in the main text, which forms the denominator of the ratio R (Eq (7)). With inclusion of the drop rate, this ratio should be close to 1 for a true β-sharing pair; neglecting dropping shifts this ratio to higher values, as seen in the left-hand cluster in Figure G, but discrimination of β-sharing and dual TCRα is still possible. The computational limitation on the calculation of multinomial coefficients does not exist for wells with smaller sample sizes, and so likelihoods can be directly calculated for clones that appear in these smaller wells. This approach is discussed in Section 6 of S1 Text.

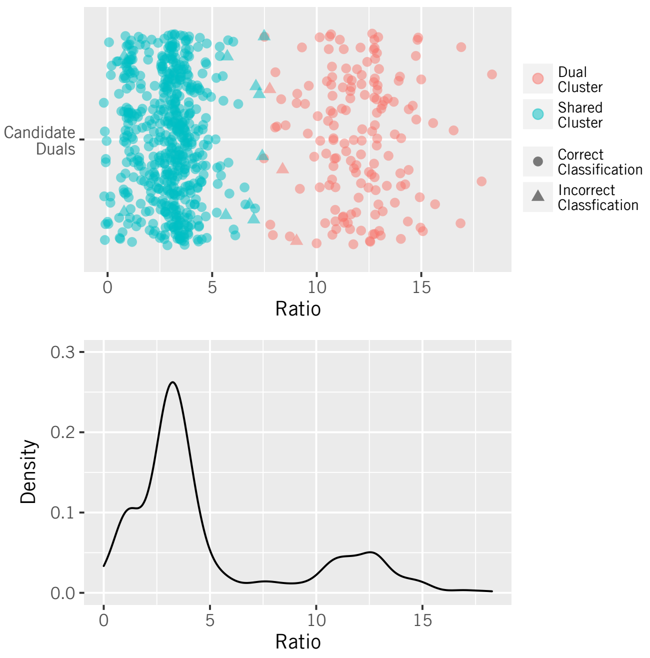

Figure G: **Discriminating between β-sharing and dual TCRα clones.** For each candidate β-sharing clone pair ($\alpha_1\beta$, $\alpha_2\beta$) we calculate the ratio of the observed number of co-occurences of the three chains to the number expected under the hypothesis they are indeed distinct clones. The latter uses the estimated frequencies of the two clones. These ratios typically partition into a lower set of values that represent β sharers and those in which co-occurrences are more common (dual TCRα). Note that the lower cluster is not centred on one; this is because calculating the expected number of co-occurrences of all three chains under the two hypotheses with a non-zero drop rate ϵ is computationally intractable due to large multinomial coefficients. However, assuming $\epsilon = 0$ biases both estimates, and k-means still resolves the two clusters of ratios.

6 Calculation of likelihoods of two- and three-way co-occurrences of chains under the hypotheses of TCRβ-sharing and dual TCRα

Discriminating between relatively abundant β-sharing clones and dual TCRα clones requires comparing the likelihoods of the data under these two hypotheses. Let $\alpha_q\beta$ and $\alpha_r\beta$ be a pair of candidate TCRs that share the same β chain with frequencies f_q and f_r respectively. Given the data have wells of s distinct sample sizes, we record the numbers of wells of each sample size that contain all three chains (α_q, α_r, β) or contain only two of the three.

For $i \in \{1,\ 2,\ \ldots,\ s\}$, let

$k_i^1 =$ the number of wells of sample size n_i containing chains β and α_q only

$k_i^2 =$ be the number of wells of sample size n_i containing chains β and α_r only

$k_i^3 =$ be the number of wells of sample size n_i containing chains α_q and α_r only

$k_i^d =$ be the number of wells of sample size n_i containing all three chains β, α_q, and α_r

$k_i^o = w_i - k_i^1 - k_i^2 - k_i^3 - k_i^d$ be the number of wells of sample size n_i that contain none of the chains or only one of the three chains.

For chains a, b, and c, let W^i_{abc}, W^i_{ab}, W^i_a, and $W^i_\emptyset$ denote the events of finding exactly chains a, b, c, finding exactly chains a and b, finding exactly chain a, and finding none of the chains in a well of sample size n_i respectively. As before, let w_i denote the number of wells with sample size n_i cells per well, and let s be the number of distinct sample sizes.

The likelihood of observing the data $\mathcal{K} = \{k_i^1, k_i^2, k_i^3, k_i^d, k_i^o : k = 1, 2, \ldots, s\}$ under the hypothesis that $\alpha_q\beta$ and $\alpha_r\beta$ represent two β-sharing clones is

$$\begin{aligned}
&\mathcal{L}(\mathcal{K} | \text{clone } \alpha_q\beta \text{ with frequency } f_q, \text{ clone } \alpha_r\beta \text{ with frequency } f_r) = \\
&\prod_{i=1}^{s} \frac{w_i!}{k_i^1!\, k_i^2!\, k_i^3!\, k_i^d!\, k_i^o!} P(W^i_{\alpha_q\beta})^{k_i^1} P(W^i_{\alpha_r\beta})^{k_i^2} P(W^i_{\alpha_q\alpha_r})^{k_i^3} P(W^i_{\alpha_q\alpha_r\beta})^{k_i^d} \times \\
&P(W^i_{\alpha_q} \cup W^i_{\alpha_r} \cup W^i_\beta \cup W^i_\emptyset)^{k_i^o}
\end{aligned} \tag{6.1}$$

where

$$
\begin{aligned}
P(W^i_{\alpha_q\beta}) =& \sum_{k=1}^{n_i} \binom{n_i}{k} f_q^k \left(1 - f_q - f_r\right)^{n_i-k} \left(1-\epsilon^k\right)^2 + \\
& \sum_{n_1=1}^{n_i-1} \sum_{n_2=1}^{n_i-n_1} \frac{n_i!}{n_1!\,n_2!\,(n_i-n_1-n_2)!} f_q^{n_1} f_r^{n_2} \left(1 - f_q - f_r\right)^{n_i-n_1-n_2} \left(1-\epsilon^{n_1}\right)^2 \epsilon^{n_2} + \\
& \sum_{n_1=1}^{n_i-1} \sum_{n_2=1}^{n_i-n_1} \frac{n_i!}{n_1!\,n_2!\,(n_i-n_1-n_2)!} f_q^{n_1} f_r^{n_2} \left(1 - f_q - f_r\right)^{n_i-n_1-n_2} \left(1-\epsilon^{n_1}\right) \epsilon^{n_1} \left(1-\epsilon^{n_2}\right) \epsilon^{n_2} \\
P(W^i_{\alpha_r\beta}) =& \sum_{k=1}^{n_i} \binom{n_i}{k} f_r^k \left(1 - f_q - f_r\right)^{n_i-k} \left(1-\epsilon^k\right)^2 + \\
& \sum_{n_2=1}^{n_i-1} \sum_{n_1=1}^{n_i-n_2} \frac{n_i!}{n_1!\,n_2!\,(n_i-n_1-n_2)!} f_r^{n_1} f_q^{n_2} \left(1 - f_q - f_r\right)^{n_i-n_1-n_2} \left(1-\epsilon^{n_2}\right)^2 \epsilon^{n_1} + \\
& \sum_{n_2=1}^{n_i-1} \sum_{n_1=1}^{n_i-n_2} \frac{n_i!}{n_1!\,n_2!\,(n_i-n_1-n_2)!} f_r^{n_1} f_q^{n_2} \left(1 - f_q - f_r\right)^{n_i-n_1-n_2} \left(1-\epsilon^{n_1}\right) \epsilon^{n_1} \left(1-\epsilon^{n_2}\right) \epsilon^{n_2} \\
P(W^i_{\alpha_q\alpha_r}) =& \sum_{n_1=1}^{n_i-1} \sum_{n_2=1}^{n_i-n_1} \frac{n_i!}{n_1!\,n_2!\,(n_i-n_1-n_2)!} f_q^{n_1} f_r^{n_2} \left(1 - f_q - f_r\right)^{n_i-n_1-n_2} \epsilon^{n_1} \left(1-\epsilon^{n_1}\right) \epsilon^{n_2} \left(1-\epsilon^{n_2}\right) \\
P(W^i_{\alpha_q\alpha_r\beta}) =& \sum_{n_1=1}^{n_i-1} \sum_{n_2=1}^{n_i-n_1} \frac{n_i!}{n_1!\,n_2!\,(n_i-n_1-n_2)!} f_q^{n_1} f_r^{n_2} \left(1 - f_q - f_r\right)^{n_i-n_1-n_2} \epsilon^{n_1} \left(1-\epsilon^{n_1}\right) \left(1-\epsilon^{n_2}\right)^2 + \\
& \sum_{n_1=1}^{n_i-1} \sum_{n_2=1}^{n_i-n_1} \frac{n_i!}{n_1!\,n_2!\,(n_i-n_1-n_2)!} f_q^{n_1} f_r^{n_2} \left(1 - f_1 - f_2\right)^{n_i-n_1-n_2} \left(1-\epsilon^{n_1}\right)^2 \left(1-\epsilon^{n_2}\right)^2 + \\
& \sum_{n_1=1}^{n_i-1} \sum_{n_2=1}^{n_i-n_1} \frac{n_i!}{n_1!\,n_2!\,(n_i-n_1-n_2)!} f_q^{n_1} f_r^{n_2} \left(1 - f_q - f_r\right)^{n_i-n_1-n_2} \left(1-\epsilon^{n_1}\right)^2 \epsilon^{n_2} \left(1-\epsilon^{n_2}\right) \\
P(W^i_{\alpha_q} \cup W^i_{\alpha_r} \cup W^i_{\beta} \cup W^i_{\emptyset}) =& 1 - P(W^i_{\alpha_q\beta}) - P(W^i_{\alpha_r\beta}) - P(W^i_{\alpha_q\alpha_r}) - P(W^i_{\alpha_q\alpha_r\beta})
\end{aligned}
$$

The likelihood of observing the data $\mathcal{K} = \{k_i^1, k_i^2, k_i^3, k_i^d, k_i^o : k = 1, 2, \ldots, s\}$ under the hypothesis that $\alpha_q\beta$ and $\alpha_r\beta$ represent one dual TCR clone is

$$
\begin{aligned}
&\mathcal{L}(\mathcal{K} | \text{clone } \alpha_q\alpha_r\beta \text{ with freq } f_d) = \\
&\prod_{i=1}^{s} \frac{w_i!}{k_i^1!\,k_i^2!\,k_i^3!\,k_i^d!\,k_i^o!} P(W^i_{\alpha_q\beta})^{k_i^1} P(W^i_{\alpha_r\beta})^{k_i^2} P(W^i_{\alpha_q\alpha_r})^{k_i^3} P(W^i_{\alpha_q\alpha_r\beta})^{k_i^d} \\
&P(W^i_{\alpha_q} \cup W^i_{\alpha_r} \cup W^i_{\beta} \cup W^i_{\emptyset})^{k_i^o}
\end{aligned}
\tag{6.2}
$$

where

$$
\begin{aligned}
P(W^i_{\alpha_q\beta}) = P(W^i_{\alpha_r\beta}) = P(W^i_{\alpha_q\alpha_r}) &= \sum_{k=1}^{n_i} \binom{n_i}{k} f_d^k \left(1 - f_d\right)^{n_i-k} \epsilon^k \left(1-\epsilon^k\right)^2 \\
P(W^i_{\alpha_q\alpha_r\beta})^{k_i^d} &= \sum_{k=1}^{n_i} \binom{n_i}{k} f_d^k \left(1 - f_d\right)^{n_i-k} \left(1-\epsilon^k\right)^3 \\
P(W^i_{\alpha_q} \cup W^i_{\alpha_r} \cup W^i_{\beta} \cup W^i_{\emptyset}) &= 1 - P(W^i_{\alpha_q\beta}) - P(W^i_{\alpha_r\beta}) - P(W^i_{\alpha_q\alpha_r}) - P(W^i_{\alpha_q\alpha_r\beta})
\end{aligned}
$$

The derivation of $P(W^i_{\alpha_q\beta})$ term for the two β-sharing clone hypothesis is shown below, and the other terms can be derived in a similar fashion. We begin by writing down the events that would result in a well containing the chains α_q and β exactly (illustrated in Figure H):

- A_i: clone $\alpha_q\beta$ is sampled in the well w_i and at least 1 α_q and β not dropped from clone $\alpha_q\beta$, clone $\alpha_r\beta$ not sampled
- B_i: clone $\alpha_q\beta$ is sampled in the well w_i and at least 1 α_q and β not dropped from clone $\alpha_q\beta$, clone $\alpha_r\beta$ is sampled and all α_r are dropped and at least 1 β not dropped from clone $\alpha_r\beta$
- C_i: clone $\alpha_q\beta$ is sampled in the well w_i and at least 1 α_q not dropped and all β dropped from clone $\alpha_q\beta$, clone $\alpha_r\beta$ is sampled and all α_r are dropped and at least one β not dropped

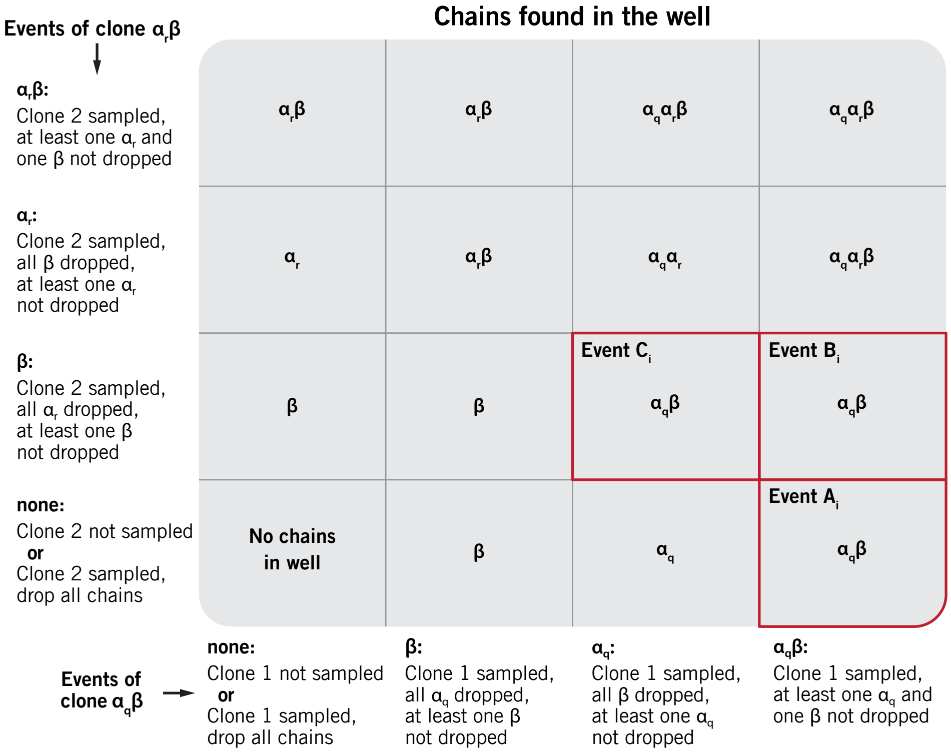

Figure H: **Sample space for calculating likelihoods of two- and three-way co-occurrences of chains under the hypotheses of TCRβ-sharing.** The events labeled in red represent all of the possible ways a well could contain the chains α_q and β from two β-sharing clones $\alpha_q\beta$ and $\alpha_r\beta$.

For event A_i, we first calculate the probability of the two independent events of (i) sampling clone $\alpha_q\beta$ without sampling clone $\alpha_r\beta$ and (ii) not dropping all of the α_q and β chains of the sampled $\alpha_q\beta$ clone. For the former, we calculate the probability of sampling clone $\alpha_q\beta_i$ from 1 to n_i times while not sampling $\alpha_r\beta_i$. Each of n_i cells in the well has a probability of f_q of being clone $\alpha_q\beta_i$ and a probability $1 - f_q - f_r$ of not being clone $\alpha_q\beta_i$ or $\alpha_r\beta_i$, which follows a binomial distribution. For the latter, each chain has a probability ϵ of being dropped, so if i cells are sampled as clone $\alpha_q\beta$, then the probability of dropping all α_q chains from those i cells is $1 - \epsilon^i$ (and similarly $1 - \epsilon^i$ for the β chains. Combining these, we get

$$\sum_{k=1}^{n_i} \binom{n_i}{k} f_q^k \left(1 - f_q - f_r\right)^{n_i - k} \left(1 - \epsilon^k\right)^2$$

We then calculate the probability of (i) sampling clone $\alpha_q\beta$ n_1 times, sampling clone $\alpha_r\beta$ n_2 times, and sampling any other clone $n_i - n_1 - n_2$ times, (ii) not dropping all of the α_q and β chains of the sampled $\alpha_q\beta$ cells, and (iii) dropping all of the α_r and β chains of the sampled $\alpha_r\beta$ cells. This is a multinomial distribution of the three sampling events multiplied by the probability of not dropping all

of the chains of clone $\alpha_q\beta$ while dropping all of the chains of $\alpha_r\beta$, which is

$$\sum_{n_1=1}^{n_i-1}\sum_{n_2=1}^{n_i-n_1}\frac{n_i!}{n_1!\,n_2!\,(n_i-n_1-n_2)!}f_q^{n_1}f_r^{n_2}\left(1-f_q-f_r\right)^{n_i-n_1-n_2}\left(1-\epsilon^{n_1}\right)^2\epsilon^{2n_2}$$

Adding these two together, we get

$$P(W^i_{\alpha_q\beta})=\sum_{k=1}^{n_i}\binom{n_i}{k}f_q^k\left(1-f_q-f_r\right)^{n_i-k}\left(1-\epsilon^k\right)^2+$$
$$\sum_{n_1=1}^{n_i-1}\sum_{n_2=1}^{n_i-n_1}\frac{n_i!}{n_1!\,n_2!\,(n_i-n_1-n_2)!}f_q^{n_1}f_r^{n_2}\left(1-f_q-f_r\right)^{n_i-n_1-n_2}\left(1-\epsilon^{n_1}\right)^2\epsilon^{2n_2}$$

For event B, we calculate the probability of n_1 cells being sampled as $\alpha_q\beta$, n_2 cells being sampled as $\alpha_r\beta$, and $n_i-n_1-n_2$ cells being sampled as neither of the two clones, where $n_1\geq 1, n_2\geq, n_1+n_2\leq n$. This looks like a multinomial distribution of three events with probabilities f_q, f_r, and $1-f_q-f_r$ occurring n_1, n_2, and $n_i-n_1-n_2$ times. This is multiplied by the probability of not dropping all of the chains from the n_1 cells of clone $\alpha_q\beta$, not dropping all of the β chains from the n_2 cells of clone $\alpha_r\beta$, and dropping all α_q chains from the n_2 cells of clone $\alpha_r\beta$. Then

$$\sum_{n_1=1}^{n_i-1}\sum_{n_2=1}^{n_i-n_1}\frac{n_i!}{n_1!\,n_2!\,(n_i-n_1-n_2)!}f_q^{n_1}f_r^{n_2}\left(1-f_q-f_r\right)^{n_i-n_1-n_2}\left(1-\epsilon^{n_1}\right)^2\epsilon^{n_2}\left(1-\epsilon^{n_2}\right) \quad (6.3)$$

For event C, we calculate a similar multinomial probability as above and multiply it by the probability of dropping all β chains from the n_1 cells of clone $\alpha_q\beta$, not dropping all α_q chains from the n_1 cells of clone $\alpha_q\beta$, dropping all of the α_r chains from n_2 cells of clone $\alpha_r\beta$, and not dropping all of the β chains from the n_2 cells of clone $\alpha_r\beta$. From this,

$$\sum_{n_1=1}^{n_i-1}\sum_{n_2=1}^{n_i-n_1}\frac{n_i!}{n_1!\,n_2!\,(n_i-n_1-n_2)!}f_q^{n_1}f_r^{n_2}\left(1-f_q-f_r\right)^{n_i-n_1-n_2}\left(1-\epsilon^{n_1}\right)\epsilon^{n_1}\left(1-\epsilon^{n_2}\right)\epsilon^{n_2} \quad (6.4)$$

Since $P(W^i_{\alpha_q\beta})=P(A_i)+P(B_i)+P(C_i)$, we add all three expressions to obtain the term as stated above.

We assessed empirically that if the difference between the logarithms of the likelihoods in Eq (6.2) and Eq (6.1) is greater than or equal to 10, then chains α_q, α_r, and β should be assumed to comprise a dual TCRα clone. As noted before, the multinomial coefficients contained in these equations are computationally limiting for wells of large sample sizes, and so calculations of these likelihoods include only wells of sample sizes less than 50 cells per well. Since these wells are most likely to contain the common clones, this approach is applicable to distinguishing common β-sharing and dual TCRα clones.

7 Comparison of depths achieved by single-cell sequencing and ALPHABETR for populations with different clonal size distributions

We compared the depths achieved by single-cell sequencing and ALPHABETR by sampling cells from the same synthetic T cell populations. In addition to the comparison discussed in the main text (using a population of 2100 clones with 25 clones comprising the top 50%), we explored different skewnesses – that is, populations of 2100 clones with 5, 10, and 50 clones comprising the top 50% (Figure I). All three sets of simulations recapitulate our conclusions that substantially larger numbers of samples need to be sequenced with single-cell methods to match the depths achieved by ALPHABETR, particularly for rare clones.

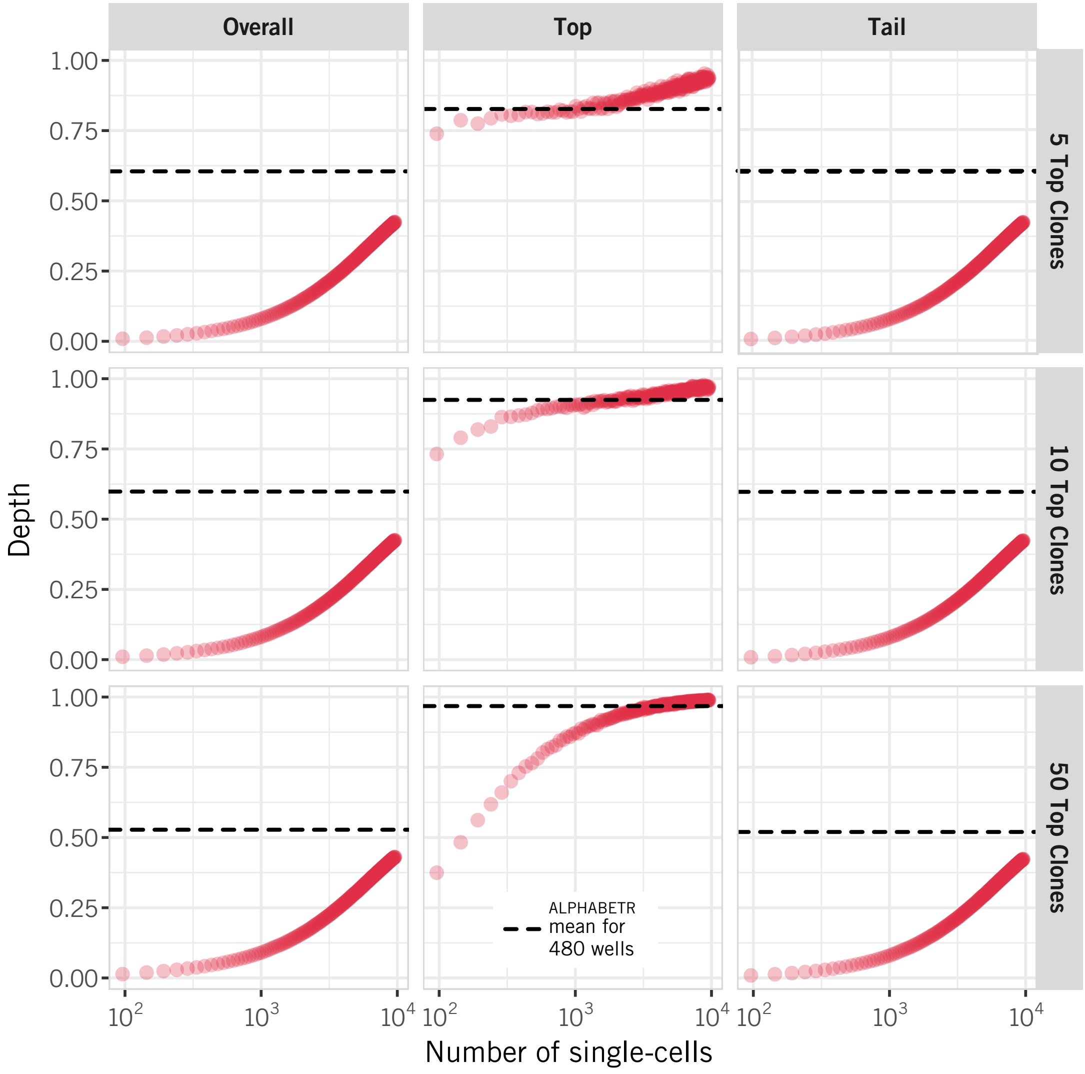

Figure I: **Comparison of single-cell approaches and ALPHABETR for different clonal size distributions.** Similar simulations shown in Fig 6 in the main text are shown here for populations of 2100 clones with 5, 10, and 50 clones making up the top 50% in frequency. The results were evaluated for top depth, tail depth, and overall depth. The dashed lines show the mean performance of ALPHABETR with 5 plates using the high-mixed sampling strategy and a threshold of 0.6 (values taken from Figure 3 in the main text). The single-cell sequencing results are averages of 200 simulations.

8 Applying ALPHABETR to TCR sequencing data from tumour-infiltrating lymphocyte populations

The TIL sequencing data described by Howie *et al.* [4] provided an opportunity to test ALPHABETR on a real TCR sequencing dataset of restricted diversity. The data were obtained by sampling T cells from nine different tumour samples into one 96-well plate and determining the CDR3α and CDR3β sequences in each well. In addition, bulk sequencing of the CDR3α and CDR3β sequences was performed on PMBCs from each patient. This allowed for the association of a subset of the sequences found in the mixed 96-well plate with their tumour sources.

Because the mixed plate contained 561452 unique CDR3α nucleotide sequences and 955987 unique CDR3β nucleotide sequences, these data are too diverse for direct input into ALPHABETR. We therefore made tumour-specific virtual plates by matching CDR3 regions in the wells to the libraries of CDR3 sequences obtained from bulk sequencing of PMBC sampled from each patient. This was done by exact matching of the first 76 bases of the nucleotide sequences of CDR3α pairSEQ sequences and the last 76 bases of the nucleotide sequences of the CDR3α libraries of each tumour sample. For the CDR3β, matching regions were 81 bases in length. These choices reflect the different reads utilised by pairSEQ and immunoSEQ sequencing. Each plate of tumour-specific chains was then analysed using ALPHABETR to obtain $\alpha\beta$ pairs.

We compared the pairs from ALPHABETR to those pairs determined in ref. [4] for which both the CDR3α and CDR3β chains were explicitly associated with exactly one tumour. Howie *et al.* also reported clones for which only one chain was associated with a tumour and the other was not found in any of the samples. Since ALPHABETR was applied only to the chains from the mixed plate that were definitively associated with one tumour, we removed the partially-matched clones called in ref. [4] from our comparative analysis.

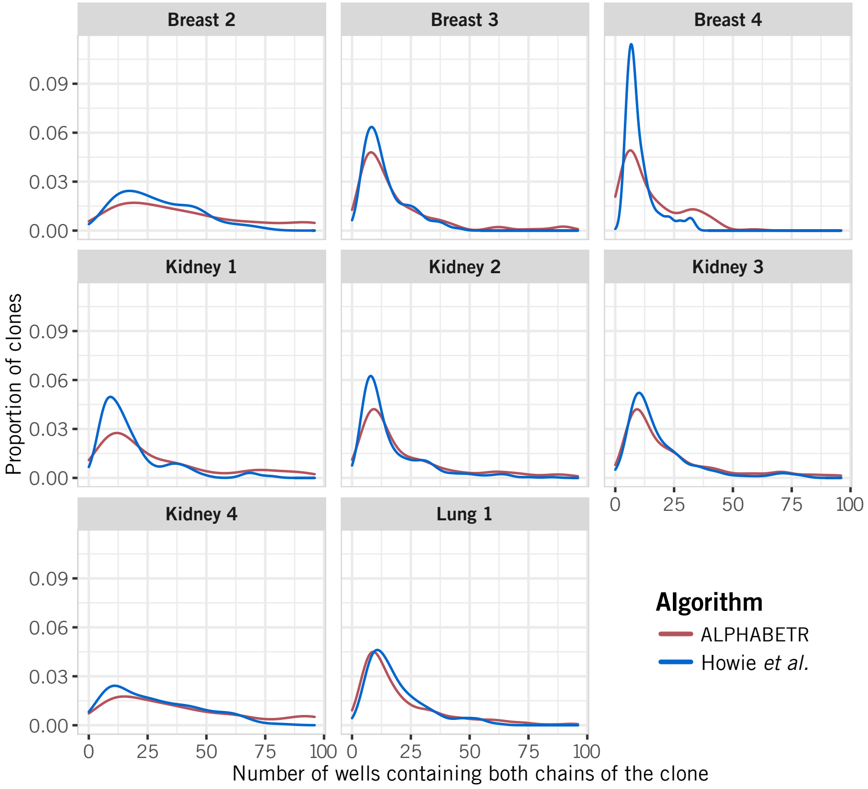

Figure J: **Tumour-specific comparisons of the levels of well occupancy of clones identified from the TIL data from ref. [4], using their method and using ALPHABETR.** The global trends seen in Figure 7 in the main text are recapitulated in these tumour-level comparisons; under the sampling strategy used in ref. [4], ALPHABETR is less efficient at identifying rare clones, and slightly more efficient at identifying clones present in more than 25% of the wells.

References

[1] Murugan A, Mora T, Walczak AM, Callan CG Jr. Statistical inference of the generation probability of T-cell receptors from sequence repertoires. Proc Natl Acad Sci U S A. 2012;109(40):16161–6. doi:10.1073/pnas.1212755109.

[2] Desponds J, Mora T, Walczak AM. Fluctuating fitness shapes the clone-size distribution of immune repertoires. Proc Natl Acad Sci U S A. 2016;113(2):274–9. doi:10.1073/pnas.1512977112.

[3] Qi Q, Liu Y, Cheng Y, Glanville J, Zhang D, Lee JY, et al. Diversity and clonal selection in the human T-cell repertoire. Proc Natl Acad Sci U S A. 2014;111(36):13139–44. doi:10.1073/pnas.1409155111.

[4] Howie B, Sherwood AM, Berkebile AD, Berka J, Emerson RO, Williamson DW, et al. High-throughput pairing of T cell receptor α and β sequences. Sci Transl Med. 2015;7(301):301ra131. doi:10.1126/scitranslmed.aac5624.
